# Nitrogen, Oxygen‐Codoped Vertical Graphene Arrays Coated 3D Flexible Carbon Nanofibers with High Silicon Content as an Ultrastable Anode for Superior Lithium Storage

**DOI:** 10.1002/advs.202104685

**Published:** 2022-01-06

**Authors:** Yongbiao Mu, Meisheng Han, Buke Wu, Yameng Wang, Zhenwei Li, Jiaxing Li, Zheng Li, Shuai Wang, Jiayu Wan, Lin Zeng

**Affiliations:** ^1^ Department of Mechanical and Energy Engineering Southern University of Science and Technology Shenzhen 518055 China; ^2^ Songshan Lake Materials Laboratory Dongguan Guangdong 523808 China; ^3^ Key Laboratory of Energy Conversion and Storage Technologies Southern University of Science and Technology Ministry of Education Shenzhen 518055 China

**Keywords:** carbon nanofibers, free‐standing and foldable electrodes, lithium‐ion batteries, Si anode, vertical graphene arrays

## Abstract

Free‐standing and foldable electrodes with high energy density and long lifespan have recently elicited attention on the development of lithium‐ion batteries (LIBs) for flexible electronic devices. However, both low energy density and slow kinetics in cycling impede their practical applications. In this work, a free‐standing and binder‐free N, O‐codoped 3D vertical graphene carbon nanofibers electrode with ultra‐high silicon content (VGAs@Si@CNFs) is developed via electrospinning, subsequent thermal treatment, and chemical vapor deposition processes. The as‐prepared VGAs@Si@CNFs electrode exhibits excellent conductivity and flexibility because of the high graphitized carbon nanofiber network and abundant vertical graphene arrays. Such 3D all‐carbon architecture can be fabulous for providing a conductive and mechanically robust network, further improving the kinetics and restraining the volume expansion of Si NPs, especially with an ultra‐high Si content (>90 wt%). As a result, the VGAs@Si@CNFs composite demonstrates a superior specific capacity (3619.5 mAh g^−1^ at 0.05 A g^−1^), ultralong lifespan, and outstanding rate capability (1093.1 mAh g^−1^ after 1500 cycles at 8 A g^−1^) as a free‐standing anode for LIBs. It is believed that this work offers an exciting method for developing free‐standing and high‐energy‐density electrodes for other energy storage devices.

## Graphical abstract

A free‐standing ultra‐high silicon content flexible anode with enhanced hydrophilicity is successfully developed by growing vertical graphene arrays on Si@CNFs (VGAs@Si@CNFs), which is regarded as “building blocks” and “bridges” to improve the conductivity the electrode, prevent the aggregation and pulverization of Si. This work casts new light on the high‐energy anode design for possessing rate capability and cycling stability.

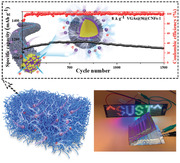

## 1 Introduction

In order to satisfy the ever‐increasing requirements for electronics, electric vehicles, and smart grid storage, rechargeable batteries with advanced electrodes have been rapidly developed for energy dense storage systems.^[^ ^1^, ^2^, ^3^, ^4^ ^]^ Lithium‐ion batteries (LIBs) as recognized as the pillar of energy storage and conversion owing to their high energy density, high work voltage, abundant resources, and long cycling lifespan.^[^ ^5^, ^6^, ^7^, ^8^, ^9^, ^10^ ^]^ As is well known, the electrochemical performance of LIBs is still primarily restricted by electrode materials, especially the anode materials.^[^ ^11^, ^12^, ^13^, ^14^ ^]^ Among various anode materials, silicon (Si) has attracted considerable attentions as one of the most promising anode material for the next generation of LIBs due to its high theoretical specific capacity of 4200 mAh g^−1^ (corresponding to Li_4.4_Si), low electrochemical potential of Li insertion/extraction (<0.5 V versus Li/Li^+^), natural abundance, and environmentally benignity.^[^ ^15^, ^16^, ^17^, ^18^ ^]^ Unfortunately, Si anode suffered from several critical challenges that have seriously limited their practical applications. 300% volume change of Si during repetitive electrochemical cycling causes severe pulverization, unstable solid‐electrolyte interphase (SEI) formation, and loss of electrical contact, which resulting in rapid capacity fading, poor rate capability, and limited cycle life.^[^ ^19^, ^20^, ^21^, ^22^ ^]^

Up to date, tremendous efforts have been made on rationally designing and fabricating novel Si‐based anode materials that display long cycle life without sacrificing energy density. The strategies to improve the electrochemical performance of Si‐based composites include: i) design and synthesis of various nanostructured materials, including 0D (nanoparticles,^[^ ^23^, ^24^ ^]^ nanospheres),^[^ ^18^, ^22^ ^]^ 1D (nanowires^[^ ^25^ ^]^ and nanotubes^[^ ^17^ ^]^), 2D (thin film,^[^ ^26^ ^]^ nanosheets,^[^ ^27^ ^]^ and nanowalls^[^ ^28^ ^]^), and 3D (porous silicon^[^ ^15^, ^16^ ^]^), for relieving volume change of Si and shortening the ion diffusion distance; ii) incorporation with carbonaceous materials as the supporting matrix to improve the conductivity and buffer the volume expansion, such as graphite,^[^ ^19^, ^29^ ^]^ amorphous carbon,^[^ ^30^ ^]^ carbon nanotubes,^[^ ^20^ ^]^ graphene,^[^ ^23^, ^31^, ^32^, ^33^ ^]^ etc.; iii) constructing core‐shell nanostructures with short ions diffusion length, efficient electronic transport pathways, and strong volume inhibition effect.^[^ ^34^, ^35^, ^36^ ^]^ Among these methods, carbon coating has been demonstrated as an attractive choice to accommodate the volume expansion and enhance kinetics of Si. Multifarious Si–C composite nanostructures, such as Si–amorphous carbon, Si–graphene, Si–carbon nanotubes, Si–graphite, and Si–carbon nanofibers,^[^ ^37^, ^38^ ^]^ have been reported recently. Despite their significantly improved electrochemical performance, most of the previous synthetic routes still face challenges such as: a) inhomogeneous coating of carbon from the common methods, including ball milling, solution mixing, and templating method, which result in rapid decay of electrochemical stability; b) the difficulty in tuning the thickness and compactness of carbon layers causes a terrible balance between electrons conduction and ions transporting; c) low Si content for the whole electrode severely leads to low energy density and weaken the capacity advantage of Si anode. As a result, a novel and controllable carbon coating process is desired for Si anode to obtain outstanding electrochemical performance.

Electrospinning has been used as an effective strategy to develop nanofibers and nanoparticles composite for LIBs.^[^ ^39^, ^40^, ^41^, ^42^, ^43^, ^44^, ^45^ ^]^ The carbon nanofibers (CNFs) electrodes have been demonstrated high overall electrode capacities for LIBs due to binder‐free, conductive additive‐free and collector‐free. In particular, CNFs have been used to provide ample buffer space and excellent flexibility for achieving a good electrical conductivity, high specific surface area, high mechanical strength electrode, which contributes to superior electrochemical properties. Incorporation of Si nanoparticles (Si NPs) in CNFs by electrospinning has emerged as a facile and effective method for achieving high performance Si anodes.^[^ ^46^, ^47^, ^48^ ^]^ However, most of the previous reported silicon@carbon nanofibers (Si@CNFs) composite anodes show insignificant specific capacity and relatively poor cyclic stability. There are three main reasons for this: i) the large specific surface energy of Si NPs leads to serious agglomeration in the electrospinning process, so that some of the silicon nanoparticles are not wrapped by carbon nanofibers, which resulting in poor cycling stability; ii) the Si content in the electrospun Si@CNFs anode is low, which loses the intrinsic capacity advantage of the Si anode; iii) the poor mechanical strength and structural stability of the composite anode is attributed to the improper heat‐treatment process. Therefore, a composite Si@CNFs anode with high Si content, high mechanical properties and well‐dispersion Si NPs is expected to be synthesized to exhibit outstanding electrochemical performance.

Herein, for the first time, we successfully design a vertical growing strategy of graphene nanosheets arrays (VGAs) on carbon nanofibers (CNFs) with controllable content of Si NPs by combining the technologies of thermal chemical vapor deposition (thermal CVD) and electrospinning to solve the problem of Si anode. Through our strategies, a free‐standing and binder‐free nitrogen (N), oxygen (O) ‐codoped 3D vertical graphene CNFs electrode with ultra‐high Si content (VGAs@Si@CNFs) is obtained. The 3D conductive and mechanical all‐carbon networks have multiple advantages: firstly, the Si@CNFs with high Si content are designed and synthesized via an electrospinning technique and subsequent thermal treatment, realizing a free‐standing Si@CNFs composite anode for LIBs without binders or conductive additives; second, VGAs@Si@CNFs is obtained by growing vertical graphene nanosheets arrays on Si@CNFs via thermal CVD method, which presents greatly enhanced electrical conductivity of the whole electrode, alleviating the volume expansion of Si NPs, promoting the formation of stable SEI and maintaining the structural stability of electrodes; thirdly, nitrogen (N), oxygen (O) codoped VGAs with abundant exposed edges and active sites are realized by after‐treatment of oxygen (O_2_)and ammonia (NH_3_), enhancing the wettability of nanofiber matrix to electrolyte and Li ions; fourthly, carbon nanofiber and vertical graphene nanosheets arrays are interconnected into 3D network structure, in which Si NPs are tightly wrapped; in addition, it is encouraging to fabricate a free‐standing, binder‐free, current collector‐free and flexible electrode through a synthesis strategy of facile, scalable, cost‐effective, and suitable for mass production of flexible batteries. As a result of these improvements, when used as anode materials in LIBs, the present VGAs@Si@CNFs electrode delivered high specific capacity (3619.5 mAh g^−1^ at 0.05 A g^−1^), exceptional rate capability (3206.9 mAh g^−1^ at 0.1 A g^−1^ in comparison with 1064.7 mAh g^−1^ at 10 A g^−1^) and outstanding cycling stability (95% capacity retention after 1500 cycles). Furthermore, when assembled into full batteries against LiFePO_4_(LFP) or LiCoO_2_ (LCO) electrode, the VGAs@Si@CNFs composite as anode delivered excellent rate capacity and cycling lifespan. This novel structure dramatically enhances the stability and efficiently resists the volume‐change‐induced mechanical strain of Si, suggesting great development in Si‐based anode.

## 2 Result and Discussion

### 2.1 Morphological and Structural Characterizations

**Figure 1:**
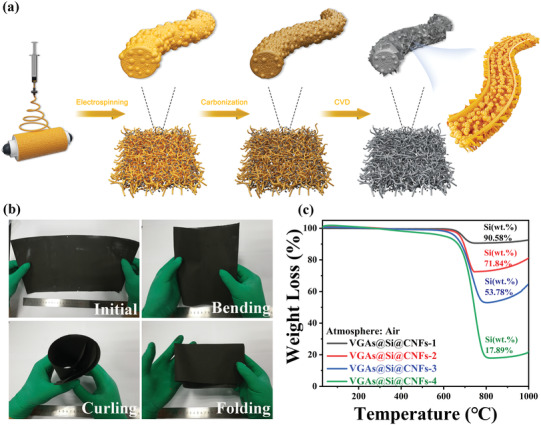
a) Schematic illustration of the fabrication process of highly flexible VGAs@Si@CNFs electrodes through the electrospinning and CVD methods. b) Photographs and demonstration of the flexible characteristic of VGAs@Si@CNFs. c) TGA curves of the as‐obtained electrodes under air atmosphere.

The detailed steps for the design and fabrication of a 3D free‐standing flexible vertical graphene carbon nanofiber film are displayed in **Figure** **1**a. At first, the primary nanostructure Si@PAN precursor was prepared by a facile electrospinning strategy using Si NPs and polyacrylonitrile (PAN) as raw materials. High voltage, stainless steel needles with larger diameter and faster injection speed were needed, which ensured high Si content and production of Si@PAN fibers. After electrospinning, a brownish Si@PAN nanofiber film was obtained (Figure S1a, Supporting Information) due to the existence of the Si NPs (Figure S1b, Supporting Information), which indicates that Si NPs are uniformly dispersed with PAN polymer. A free‐standing Si@CNFs with excellent flexibility and structural stability was successfully obtained by sequential stabilization and carbonization. It is widely known that applying tension during stabilization is crucial for preparing high‐performance CNFs.^[^ ^49^, ^50^, ^51^, ^52^ ^]^ The Si@PAN nanofiber film was stabilized through thermal‐stretching strategy at 260 ℃ for 4 h in air at a heating rate of 5 ℃ min^−1^ (Figure S2a–c, Supporting Information), the color of the fibrous film was further deepened (Figure S1c, Supporting Information). At 1000 ℃, Si@PAN polymer nanofiber is gradually carbonized under three different thermal‐stretching carbonization strategy. A high‐strength, flexible, and large area CNFs film is obtained through the biaxial thermal‐stretching (Figures S1d and S2f, Supporting Information), which is due to the stretching that could prevent the physical relaxation or shrinkage of PAN chains and improve their orientation degree.^[^ ^53^, ^54^, ^55^ ^]^ Thereafter, the VGAs@Si@CNFs film was developed through a thermal CVD process for growing VGAs on Si@CNFs. As expected, a free‐standing VGAs@Si @CNFs with a large area of more than 30 cm × 40 cm can be easily fabricated and maintained excellent mechanical flexibility with good integrity after bending, curling, and folding (Figure 1b and Video S1, Supporting Information). What's more, the VGAs@Si@CNFs‐1 electrode could be bent over 10 000 times without damage (Video S2, Supporting Information), which confirms the correctness of our biaxial thermal‐stretching strategy and potential for practical application. The various VGAs@Si@CNFs electrodes with different Si content had been fabricated by adjusting the concentration of Si NPs during the electrospinning process, which the Si content of VGAs@Si@CNFs electrodes are determined by the thermogravimetric analysis (TGA) (Figure 1d). For Si NPs, the continuous weight elevation is ascribed to oxidation of Si to SiO_2_ in air (Figure S3, Supporting Information). For CNFs and VGAs@Si@CNFs electrodes, the weight loss between 550 and 850 ℃ represents the oxidation of external carbon and internal Si NPs in air. Consequently, the calculated results of show that the Si contents of VGAs@Si@CNFs‐*X* (*X* = 1, 2, 3, 4) are 90.58%, 71.84%, 53.78%, and 17.89%, respectively, demonstrating an ultra‐high Si content free‐standing electrode (Table S1, Supporting Information).

**Figure 2:**
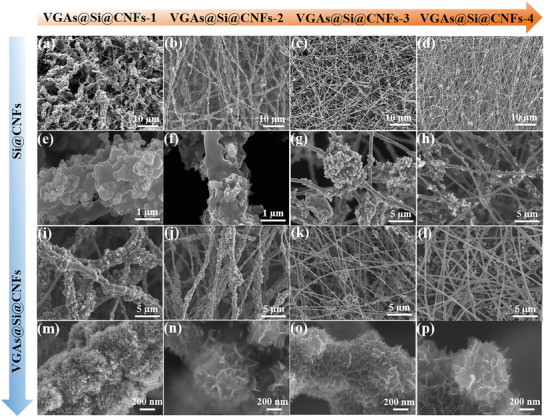
Controllable synthesis of VGAs@Si@CNFs by adjusting the concentration of Si NPs during the electrospinning process. SEM images of a,e) Si@CNFs‐1, i,m) VGAs@Si@CNFs‐1, b,f) Si@CNFs‐2, j,n) VGAs@Si@CNFs‐2, c,g) Si@CNFs‐3, k,o) VGAs@Si@CNFs‐3, d,h) Si@CNFs‐4, and l,p) VGAs@Si@CNFs‐2.

Scanning electron microscope (SEM) was used to study morphology and structural change during carbonization and thermal CVD process. SEM images reveal that the various Si@CNFs films have 1D continuous and uniform nanofiber morphology with diameters in the range of 0.5∼2.5 µm corresponding to different Si content (**Figure** **2**a–d). For the Si@CNFs‐1 electrode, the surface of fibers is exceptionally rough due to the accumulation of a large number of Si NPs (Figure 2e), compared with other Si@CNFs (Figure 2f–h) and CNFs electrodes (Figure S4a,b, Supporting Information). The result was caused by the Si NPs anchored and embedded in the fibers, occupying certain space and leading to a thicker diameter distribution (Figure S5a–d, Supporting Information). Specifically, the fibers diameters of Si@CNFs were calculated to be around 2.70, 1.50, 0.67, and 0.36 µm, which are 5.0, 2.8, 1.2, and 0.7 times as that of the original CNFs (Figure S4c, Supporting Information), respectively. The cross‐section SEM images of Si@CNFs‐1 indicate that the thickness of the electrodes is controllable by adjusting the time of electrospinning in the range of 2–6 h (Figure S6, Supporting Information). The 1D Si@CNFs are disorderly arranged into a 3D, porous, interconnected network, which provide a fast electronic and ionic pathway, contributing to enhanced kinetics. During a subsequent thermal CVD process, the direct growth of vertical graphene nanosheets arrays on Si@CNFs was achieved by introducing ethanol vapor as the carbon source via our self‐made equipment (Figure S7, Supporting Information). The VGAs evenly grown on various Si@CNFs composite by adjusting the concentrations of H_2_ and ethanol vapor (Figure 2i–l). A 3D conductive network is formed by the interconnected VGAs (Figure 2m–p), which can provide good conductive paths. The mechanism of vertical graphene growth by thermal CVD is briefly analyzed.^[^ ^56^, ^57^ ^]^ Vertical graphene usually takes a long time to grow on a flat substrate because the carbon atoms tend to curl up continuously on the flat substrate, and only when hydrogen is introduced, the carbon atoms are arranged in irregular steps and defects, and at the steps the carbon atoms are no longer arranged in the direction of the substrate, thus making the graphene grow perpendicular to the direction of the substrate (Figures S8a and S9a,b, Supporting Information). In Si@CNFs, 1D nanofibers and 0D nanoparticles have higher curvatures, which greatly increases the rate of graphene growth (Figures S8b and S9c–f, Supporting Information). Such unique interconnected structure can ensure the excellent mechanical property and stability of the electrodes during electrochemical processes.^[^ ^58^, ^59^ ^]^ The height of VGAs is about 150 nm, which can be perfectly controlled by changing the growth time (Figure S10a–d, Supporting Information). It is clear that the density and height of the vertical graphene arrays increase with extending the deposition time. In particular, the VGAs height on Si@CNFs is mainly in the range of 50∼300 nm within 3 h, while it takes more than 6 h to grow VGAs on original CNFs with a height of about 150 nm when methane (CH_4_) as carbon source (Figure S11, Supporting Information).

**Figure 3:**
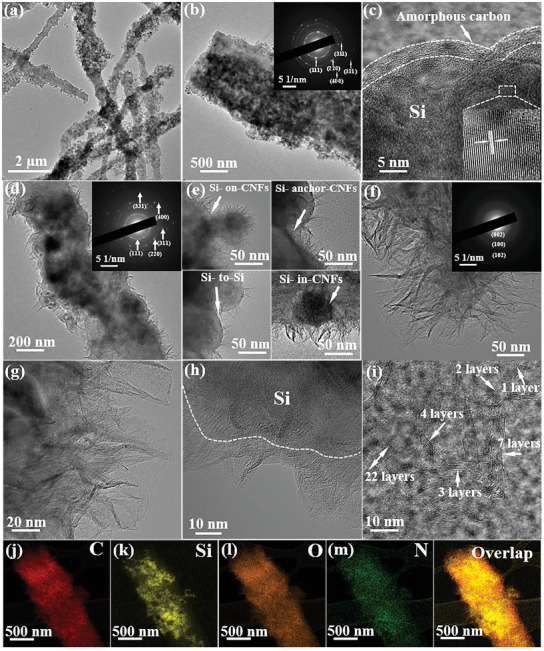
Morphology and composition characterization of Si@CNFs and VGAs@Si@CNFs: Typical HRTEM images of a–c) Si@CNFs, d–i) VGAs@Si@CNFs‐1, inset of b,d,f) SAED, and j–m) corresponding elemental mapping images of VGAs@Si@CNFs‐1.

In order to further reveal the interactions among the three components of Si NPs, CNFs, and VGAs, transmission electron microscopy (TEM) was performed. Consistent with the observation in SEM images, Si@CNFs show a stabilized fibrous morphology with a larger diameter of 0.5∼2 µm, compared with CNFs, indicating a high Si content (**Figure** **3**a and Figure S12a,b, Supporting Information). Numerous Si NPs are wrapped and anchored on the CNFs during electrospinning (Figure 3b), the Si NPs possess a crystalline structure confirmed by the selected area electron diffraction (SAED) image of Si@CNFs (inset of Figure 3b), displaying five noticeable diffraction rings that can be assigned to (311), (220), (111), (400), and (331) planes of crystal Si. The crystalline Si NPs are fully encapsulated by a PAN‐derived carbon layer with a thickness of about 3∼10 nm (Figure 3c and Figure S13a,b, Supporting Information), thus forming a continuous CNFs buffer layer, consistent with the energy dispersive spectroscopy (EDS) mapping for separate elementary mappings (Figure S14, Supporting Information). VGAs are well grown on Si@CNFs, demonstrating a height of about 150 nm (Figure 3d). From the high‐resolution TEM, positional relationships among Si NPs, CNFs, and VGAs can be summarized. On the one hand, Si NPs are anchored on the CNFs, and tightly coated by vertical graphene sheets arrays; on the other hand, Si NPs are completely wrapped in the CNFs, which are simultaneously coated by nanofibers and vertical graphene (Figure 3e). It can be clearly found that graphene nanosheets vertically grow on the surface of Si NPs (Figure 3f–h), forming a seamlessly coating layer on the well‐crystallized Si NPs. In detail, the VGAs exhibit a few‐layer structure and a top of triangle shapes (Figure 3i), demonstrating a well‐defined layered structure with an interlayer distance of 0.35 nm, coincident with the characteristic value of graphene.^[^ ^60^, ^61^ ^]^ Abundant connections are constructed by the VGAs on CNFs and Si NPs, which can increase electrical contacts and prevent the disintegration during cycling. The EDS mapping for separate elementary mappings (Figure 3j–m) further elaborates the key relationship of the VGAs, Si NPs, and CNFs: the inner Si NPs are well confined by the CNFs and VGAs layers, which not only facilitates satisfactory electron conduction but also effectively alleviate stress/strain induced by the volume change, achieving long cycle life and rate capability.

**Figure 4:**
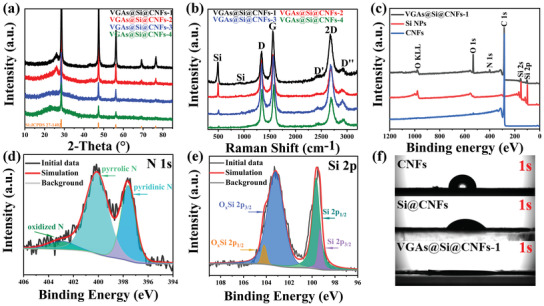
Structural and compositional analysis of various electrodes. a) XRD patterns, b) Raman spectrum, c) survey XPS spectrum with high‐resolution XPS spectra of d) N 1s, e) Si 2p, and f) CA for electrolyte (LiPF_6_) and electrode.

X‐ray diffraction (XRD), Raman, Brunauer–Emmett–Teller, and X‐ray photoelectron spectroscopy (XPS) are performed to explore the phase, defect, porous structure, and chemical composition. The original CNFs show a broad diffraction peak located at around 24.3°, corresponding to the (002) plane of amorphous carbon (Figure S15, Supporting Information). For commercial Si NPs, it exhibits sharp diffraction peaks at 28.6°, 47.7°, 56.2°, 69.4°, and 76.4°, which are respectively attributed to (111), (220), (311), (400), and (331) planes of Si crystals.^[^ ^38^, ^39^ ^]^ Hence, the XRD patterns of VGAs@Si@CNFs demonstrate that all characteristic diffraction peaks are well‐matched with planes of crystalline Si and CNFs, indicating good integration among Si NPs, CNFs, and VGAs (**Figure** **4**a), which is in accordance with our SEM and TEM observation. Raman spectroscopy is employed to characterize their carbon structure information (Figure 4b). Three prominent peaks at about 1345.9, 1578.1, and 2704.8 cm^−1,^ are assigned to D band, G band and 2D band of graphene, respectively,^[^ ^60^ ^]^ compared to the CNFs and Si@CNFs (Figure S16, Supporting Information), The intense D band is related to disorder and defects, and the D band and G band intensity ratio (*I*_D_/*I*_G_) is 0.83 for VGAs@Si@CNFs‐1, which can be attributed to the numerous edges of the VGAs, suggesting that VGAs possess vast defects beneficial for the lithium‐ion storage. The G band and 2D band intensity ratio (*I*_G_/*I*_2D_) is 1.02 for VGAs@Si@CNFs‐1, demonstrating the structure of few‐layered graphene.^[^ ^32^, ^60^ ^]^ The mechanical property of electrodes is one of the important parameters to determine the stability of electrode. The tensile stress–strain curves were used to evaluate mechanical properties of the as‐obtained electrodes (Figure S17a–c, Supporting Information). The CNFs film with biaxial stretching reaches its maximum tensile strength (∼ 6.28 MPa) at a strain of 0.054, which is better than uniaxial stretching (∼ 5.31 MPa; 0.039) and without stretching (∼ 4.53 MPa; 0.028) samples. For Si@CNFs and VGAs@Si@CNFs electrodes, the maximum tensile and strain of all Si@CNFs electrodes decrease gradually with the increase of Si content, which is due to the increase of defects in CNFs for the incorporation of Si. Fortunately, all of the electrodes with various content of Si remain highly mechanical strength after the growth of vertical graphene arrays, which meets the mechanical properties of free‐standing electrodes. Especially, the electrodes with ultrahigh mass loading of Si NPs still maintain a high integrity, good mechanical strength, and excellent flexibility because of the biaxial thermal‐stretching strategy performed during stabilization and carbonization processes. Nitrogen adsorption/desorption isotherms reveal that the VGAs@Si@CNFs‐1 has an abundant porous structure with a specific surface area of 132.2 m^2^ g^−1^, higher than those of CNFs (12.2 m^2^ g^−1^) and Si@CNFs (26.8 m^2^ g^−1^) (Figure S18a–c, Supporting Information), originating from its dense vertical graphene nanosheets arrays and post‐treatment of NH_3_ and O_2_. Abundant mesoporous structures in the range of 2∼10 nm and large pores (75∼85 nm) in VGAs@Si@CNFs‐1 networks are observed (Figure S18d, Supporting Information). Such unique structure with a characteristic pore feature effectively guarantees convenient diffusion/transport of electrolyte ions to abundant active sites of the VGAs@Si@CNFs‐1 for Li^+^ storage. The resistance of the VGAs@Si@CNFs‐1 film was measured to be 0.097 Ω cm using the four‐probe method, which is far better than that of Si NPs (2576.3 Ω cm) and CNFs (0.27 Ω cm) (Figure S19, Supporting Information). XPS was applied to investigate the surface chemistry phase and composition of the VGAs@Si@CNFs‐1. It is confirmed that the existence of C, Si, N, and O elements in the VGAs@Si@CNFs‐1 (Figure 4c). The weight contents of every element in various samples are qualified by elemental analysis (Table S2, Supporting Information). The Si contents of VGAs@Si@CNFs‐1, VGAs@Si@CNFs‐2, VGAs@Si@CNFs‐3, and VGAs@Si@CNFs‐4 are 38.36%, 27.3%, 12.14%, and 7.4%, respectively, indicating a gradual raising of Si content with increasing addition in Si@PAN precursor. The high‐resolution C 1s spectra are fitted into four peaks located at 284.7, 285.2, and 288.9 eV, corresponding to the C—C, C═N, and O═C bonds (Figure S20, Supporting Information). Three types of N species shown in N1s spectra are pyridinic N (398.3 eV), pyrrolic N (399.9 eV), and oxidized N (402.9 eV), demonstrating N‐doping of the Si@CNFs during the carbonization process of NH_3_ (Figure 4d). The Si 2p peaks centered at 99.3 and 99.8 eV belong to the bulk Si, while centered at 103.5 and 104.3 eV can be assigned to the SiO*_x_* due to carbonization and oxygen treatment (Figure 4e).^[^ ^61^ ^]^ The as‐obtained electrode wettability toward the electrolytes was validated via the contact angle (CA) measurements. The CA of VGAs@Si@CNFs‐1 is 0.8°, which is much smaller than that of Si@CNFs (52.8°) and CNFs (82.8°) electrodes after 1 s (Figure 4f). Moreover, all electrodes treated by NH_3_ and O_2_ have prominent contact with LiPF_6_ electrolyte (Figure S21, Supporting Information). This result indicates that the VGAs@Si@CNFs electrodes possess good wettability for active material and electrolyte, guaranteeing the stability and utilization of electrolyte under high Si content and loading in such free‐standing electrodes.

### 2.2 Electrochemical Performance

**Figure 5:**
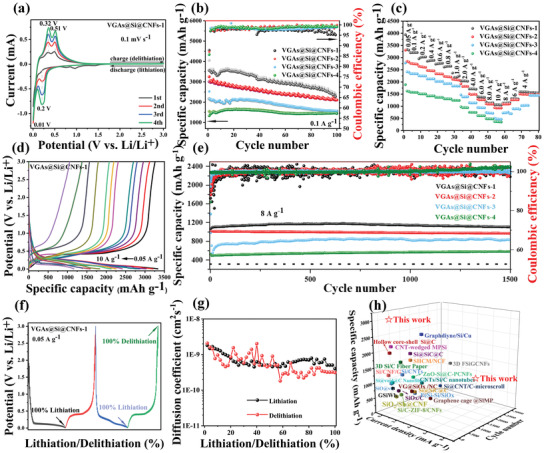
Electrochemical performances of the various VGAs@Si@CNFs electrodes as LIB anodes. a) The initial four CV curves of the VGAs@Si@CNFs‐1 electrode at 0.1 mV s^−1^. b) The cycling stability under 0.1 mA g^−1^. c) Rate capability of VGAs@Si@CNFs‐*X* (*X* = 1, 2, 3, 4) and d) charge/discharge curves of VGAs@Si@CNFs‐1 at various current densities. e) Long‐term cycling stabilities at 8 A g^−1^ of various electrodes, f) GITT profiles, and g) D_Li+_ calculated from GITT profiles. h) Performance comparison with the Si anodes modified by the prevailing method in the literature.

**Figure 6:**
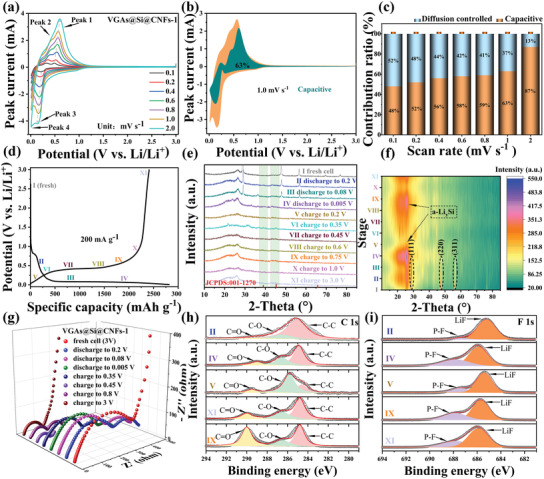
Studies on Li‐ion storage behaviors of VGAs@Si@CNFs‐1. a) The CV curves at different scan rates, b) the detailed pseudocapacitive contribution at 1 mV s^−1^, c) percentages of pseudocapacitive contributions ratios at various scan rates. d) The initial charge/discharge curves of the VGAs@Si@CNFs‐1 electrode at 0.2 A g^−1^. e) Ex situ XRD patterns at the selected depths in the discharge/charge state and f) contour plots. g) Ex situ EIS and XPS spectra of h) C 1s, i) F 1s at different charge/discharge stages.

For investigating the electrochemical performance of as‐obtained different electrode, coin‐cells were assembled using the as‐prepared electrodes as anodes and Li metal as counter electrodes. The cyclic voltammetry (CV) curves of the initial four cycles for the VGAs@Si@CNFs‐1 at 0.1 mV s^−1^ in the potential window of 0.005∼3 V are shown (**Figure** **5**a). The apparent cathodic peaks at around 0.90 and 0.01 V for VGAs@Si@CNFs‐1 electrode in the first scan, which is ascribed to the formation of SEI on VGAs coating, similar to the result published in the previous literature.^[^ ^38^ ^]^ A new reduction peak at 0.2 V appearing in the next several cycles is assigned to the lithiation reaction of Si to Li*_x_* Si alloy. Two anodic peaks located at around 0.32 and 0.51 V related to the dealloy steps of Li*_x_* Si, consistent with the CV curves of the Si@CNFs (Figure S22, Supporting Information). The alloying peak grows intensively with the cycles increasing and stabilizes at 0.2 V, confirming the gradual activation process of electrodes. According to galvanostatic charge/discharge profiles (Figure S23, Supporting Information), the stable discharge capacity of VGAs@Si@CNFs‐1 is 3233.3 mAh g^−1^ at 50 mA g^−1^, which is obviously higher than that of VGAs@Si@CNFs‐2 (2777.8 mAh g^−1^), VGAs@Si@CNFs‐3 (2350.4 mAh g^−1^), VGAs@Si@CNFs‐4 (1587.9 mAh g^−1^), respectively. Significantly, while the highest initial discharge capacity of 4080 mAh g^−1^ at 0.05 A g^−1^, Si NPs electrode delivers a lower initial coulombic efficiency (ICE) of 62.5%, which is mainly caused by the formation of SEI layers and sluggish Li‐ion transport kinetics (Figure S24, Supporting Information). A higher ICE was achieved for the VGAs@Si@CFs electrodes owing to the unique VGAs and CNFs structure, which effectively encapsulates the Si NPs inside the VGAs and CNFs, forming a double carbon coating layer. The ICE gradually decays for VGAs@Si@CNFs electrodes with the raising of Si content, which is ascribed to the high specific surface area of VGAs and high Si content. The cycle performance of VGAs@Si@CNFs (*X* = 1∼4) at 0.1 A g^−1^ in a potential range from 0.005 to 3.0 V are performed (Figure 5b). The VGAs@Si@CNFs‐1 electrode maintains the highest reversibility of 2390.5 mAh g^−1^ after 100 cycles. Moreover, VGAs@Si@CNFs electrodes (*X* = 2, 3, and 4) deliver reversible capacities of 2104.5, 1559.9, and 1451.5 mAh g^−1^ after 100 cycles, respectively, showing outstanding capacity retention of 68.9%, 73.3%, and 93.6%, respectively. It is noted that the capacity retention of the VGAs@Si@CNFs electrode with 90.58% Si is low at low current density. This phenomenon may be resulted from the difference of *x*‐value in lithiated Li*_x_* Si at different current densities. In principle, lower current density could ensure more fully lithiation of Si, thus resulting in higher *x*‐value than that at higher current density. The higher *x*‐value would bring about larger volume expansion of Si during lithiation to cause the worse stability of electrode structure, thus decreasing capacity retention. For comparison, the benchmark electrode of Si NPs suffers fast capacity fade for the subsequent cycles at a current density of 0.1 A g^−1^, which is primarily attributed to the expansion of Si during lithiation/delithiation (Figure S25, Supporting Information). Rate capabilities are subsequently conducted for evaluating the Li‐ion storage kinetics (Figure 5c). VGAs@Si@CNFs‐1 electrode exhibits outstanding reversible capacities of 3331.9, 2756.8, 2208.9, 2043.2, and 1378.3 mAh g^−1^ at 0.05, 0.6, 1, 2, and 6 A g^−1^, respectively. Excitingly, it can even acquire high reversibility of 1064.7 mAh g^−1^ at a high current density of 10 A g^−1^. All VGAs@Si@CNFs electrodes show excellent rate performance, although the capacity decreases with the increase of Si content. The associated charge/discharge profiles of VGAs@Si@CNFs‐1 electrode with different current densities are also depicted (Figure 5d). The VGAs@Si@CNFs‐1 maintains apparent voltage plateaus, even at a high‐rate up to 10 A g^−1^. The major capacity contributions originated from Si NPs are discovered below the voltage of 0.3 V through the discharge curves. Although the high specific capacities at low current densities of 50, 100, and 200 mA g^−1^, the Si NPs electrode suffers from huge capacities fading over the current density of 500 mA g^−1^ (Figure S26, Supporting Information). Importantly, excellent rate capability and cycle stability are demonstrated for CNFs and VGAs@CNFs for Li^+^ storage (Figure S27, Supporting Information). These results reveal that the excellent electrochemical properties mainly benefit from the unique VGAs grown on CNFs structure of VGAs@Si@CNFs composite, which in part synergistically derived from the long‐term continuous CNFs framework and abundant VGAs. The all‐carbon coating plays an important role in accommodating the large volume expansion of Si NPs during the charge/discharge process. To investigate the improvement of VGAs@Si@CNFs toward Si electrochemical performance, electrochemical impedance spectra (EIS) were measured (Figure S28, Supporting Information). The EIS results of the VGAs@Si@CNFs‐1 electrode before and after cycling are presented, which are fitted using the equivalent circuits (Figure S29, Supporting Information), where the meaning of the symbols is as following: *R*_s_−electrolyte resistance, corresponding to intersection of high‐frequency semicircle and *x*‐axis; *R*_f_−SEI layer resistance; *R*_ct_−charge transfer resistance, corresponding to diameter of depressed semicircle; W−Warburg impedance of Li^+^ diffusion, corresponding to slope line in low‐frequency range.^[^ ^62^ ^]^ It is obvious that the fresh VGAs@Si@CNFs‐1 electrode exhibits a low *R*_ct_ of 358.6 Ω, which even decreases to around 17.5 Ω after 100 cycles (Table S3, Supporting Information), indicating that 3D interconnected conductive network benefits for forming more stable SEI layer and boosting Li^+^ transport. The steeply inclined lines at low frequency indicate the fast lithium‐ion diffusion process within the electrode, demonstrating rapid Li^+^ ion diffusion for VGAs@Si@CNFs‐1. To better demonstrate the good lithium‐ion storage performance at high current densities, the long‐term cycling stabilities were further tracked (Figure 5e). All VGAs@Si@CNFs‐*X* (*X* = 1, 2, 3, 4) electrodes show high cycling stability up to 1500 cycles at 8 A g^−1^, corresponding to the reversible capacity of 1092.1, 960.4, 936.9, and 589.3 mAh g^−1^, which is greatly improved compared with Si NPs (Figure S30, Supporting Information). The superior cycling stability should be ascribed to the 3D continuous conductive network and the defect‐rich N, O codoped structure. To understand the influence of the Li^+^ ion diffusion on the electrode kinetics, we investigated the diffusion coefficient of Li^+^ ion using galvanostatic intermittent titration technique (GITT) methods (**Figure** **6**f,g). The Li^+^ ion diffusion coefficient ($D_{Li^{+}}$) of VGAs@Si@CNFs‐1 can be further calculated according to the simplified equations:^[^ ^37^, ^63^ ^]^

(1) $$D_{Li^{+}}=\frac{4}{\pi }{\left(\frac{m_{B}V_{M}}{M_{B}A}\right)}^{2}{\left(\frac{\Delta E_{S}}{\tau (dE_{\tau }d\sqrt{\tau }}\right)}^{2}\left(\tau \ll L^{2}/D_{Li^{+}}\right)$$

(2) $$D_{Li^{+}}=\frac{4}{\pi \tau }{\left(\frac{m_{B}V_{M}}{M_{B}A}\right)}^{2}\frac{\Delta E_{S}}{\Delta E_{\tau }}$$

where the *τ*, *V*_M_, *m*_B_, *M*_B_, *A*, Δ*E*_S_, and Δ*E*_*τ*_ stand for the titration time, the molar volume, the mass and molar mass of VGAs@Si@CNFs‐1, the surface area of the electrode, the voltage changes during the rest time, and the voltage changes during the titration time, respectively.

The results show that $D_{Li^{+}}$ value of VGAs@Si@CNFs‐1 anode located in the range of 1.7×10^−9^ ∼ 7.2×10^−10^ cm^2^ s^−1^ during the charging and discharging processes, which reveals that the Li^+^ ion diffusion in the redox reactions is greatly improved compared with the results reported in the previously published literature,^[^ ^63^ ^]^ benefiting from the 3D conductive network composed of uniform VGAs and long‐term continuous CNFs structure. To the best of our knowledge, the as‐designed VGAs@Si@CNFs electrode is competitive to those of the Si‐based materials previously reported in the literature (Figure 5h, and Table S4, Supporting Information).

### 2.3 Kinetic Evaluations and Investigations

To explain the remarkably electrochemical performance, the CV curves at various scan rates from 0.1 and 2.0 mV s^−1^ were obtained to reveal the electrochemical kinetics. The CV measurements at different sweep rates ranging from 0.1 to 2.0 mV s^−1^ are recorded to further analyze the Li^+^ (de)intercalation kinetics of VGAs@Si@CNFs‐1 anode (Figure 6a). Note that the current response (*i*) and the sweep rate (*v*) in a certain electrode can be described by the power‐law equations:^[^ ^37^, ^63^ ^]^

(3) $$i\;=\;a\;\times \;v^{b}$$

(4) $$\log\left(i\right)=\;b\log\left(v\right)+\log\left(a\right)$$

where *a* and *b* are approximate values. The *b* value is an indicator of the electrochemical behaviors, either pseudocapacitive (*b* = 1) or diffusion‐controlled (*b* = 0.5). Obviously, four similar peaks are observed in each CV curve in the VGAs@Si@CNFs‐1 electrode when the scan rate increases, including two reduction peaks and two oxidation peaks. The fitted b values of cathodic and anodic peaks are 0.73 and 0.85, indicating a mixed Li^+^ ion storage process on VGAs@Si@CNFs‐1 anode, thus leading to a fast Li^+^ ion storage kinetic (Figure S31a,b, Supporting Information). Furthermore, the specific diffusion‐controlled and pseudocapacitive‐controlled behaviors can be calculated by the following equations:

(5) $$i\;\left(V\right)=\;k_{1}v+\;k_{2}v^{1/2}$$

(6) $$i\left(V\right)/v^{1/2}=\;k_{1}v^{1/2}+k_{2}$$

where *k*_2_ *v* ^1/2^ and *k*_1_ *v* correspond to diffusion‐controlled and pseudocapacitive‐controlled behaviors, respectively. The diffusion‐controlled process plays a key role in the VGAs@Si@CNFs‐1 electrode at the scan rate of 0.1 mV s^−1^. Obviously, the pseudocapacitive contribution ratios of VGAs@Si@CNFs‐1 gradually evolves as a dominating contribution along with the scan rates increasing (0.2, 0.4, 0.6, 0.8, 1.0, and 2.0 mV s^−1^), the detailed pseudocapacitive contribution (the blue area) at 1 mV s^−1^ for VGAs@Si@CNFs‐1 are discovered (Figure 6b), and even reaches the contribution rate of 87% at 2.0 mV s^−1^ (Figure 6c), which mainly related to the unique 3D VGAs and interconnected CNFs network, which provides abundant exposed graphene edges and defects for the Li^+^ ion storage.

In order to reveal the structural, chemical stability and phase evolution of VGAs@Si@CNFs‐1 after cycling, a series of physicochemical measurements were performed, including SEM, TEM, XRD, EIS, and XPS. The VGAs@Si@CNFs‐1 electrode maintains similar morphology with the pristine electrode after cycling for 100 cycles from the SEM images (Figure S32a, Supporting Information), and the thickness of VGAs@Si@CNFs‐1 electrode after cycling was barely changed (Figure S32b, Supporting Information). C, Si, O, and N elements are evenly distributed from the cross‐sectional EDS mapping spectra before cycling (Figure S32c, Supporting Information) and after cycling (Figure S33a–f, Supporting Information), which proving robust and reliable carbon nanofiber and vertical graphene structure during the electrochemical process. The TEM images show that vertical graphene nanofibers morphology of the VGAs@Si@CNFs‐1 is well preserved (Figure S34a, Supporting Information), and the crystalline Si transforms into amorphous Si with an uniform coating of VGAs and CNFs (Figure S34b–d, Supporting Information), which demonstrates that 3D interconnected network can effectively accommodate the volume change of Si during cycling. Moreover, the elemental mapping spectra show that the C, Si, O, N elements are still uniformly distributed in the VGAs@Si@CNFs‐1, further indicating the mechanical and chemical stability of the electrode (Figure S35a–f, Supporting Information). We performed ex situ XRD and contour plots for VGAs@Si@CNFs‐1 at the different potential ranges for the first cycle at 0.2 A g^−1^ (Figure 6d). It is obvious that the Si diffraction peaks become weaker with the proceeding of reaction, the characteristic peaks of Si are fully disappeared in stage IV, indicating that crystal Si reacts with Li and amorphous Li*_x_* Si generates during the continuous lithiation process (Figure 6e,f). The involved chemical reaction follows the formula:^[^ ^37^, ^64^ ^]^ Si + *x*Li →$\left(1-\frac{x}{y}\right)$Si + $\frac{x}{y}$ a‐Li*_y_* Si, which suggests that the Si would gradually be converted into a‐Li*_y_* Si during lithiation. When *x* = *y*, the conversion of Si into a‐Li*_y_* Si is completed, which would result in the disappearance of crystalline Si. During delithiation, the chemical reaction follows the formula: a‐Li*_y_* Si→a‐Si + *y*Li, which indicates the a‐Li*_y_* Si would gradually be converted into a‐Si, corresponding to the anodic peaks from 0.005 to 1 V in the CV curves (Figure 5a). The delithiation process is also confirmed by ex situ XRD, in which Si still exists in the amorphous form, suggesting crystalline Si peaks cannot be reformed, in conformity to the previous literatures.^[^ ^21^, ^37^, ^47^ ^]^ Besides, the weak diffraction peaks of LiF as a component of SEI layer are observed at around 38.9°and 45.3°(JCPDS:001‐1270), indicating the formation of crystalline LiF.^[^ ^65^ ^]^ The formation of LiF arises from the SEI layer caused by the decomposition of electrolyte, corresponding to the cathodic peaks at around 0.90 V in the CV curves (Figure 5a). Ex situ TEM analysis was carried out to observe structural evolution of VGAs@Si@CNFs electrodes in discharge (0.005 V) and charge (3 V) position (Figure S36, Supporting Information). It is noted that the VGAs@Si@CNFs electrode maintains its fibrous structure well without any structural cracks (Figure S36a,b,e,f, Supporting Information) and the structure of VGAs is basically unchanged (Figure S36c,g, Supporting Information) when discharged to 0.005 V and charged to 3.0 V, which further confirm that the electrode structure is highly stable during cycling. SAED patterns of the full lithiation (Figure S36d, Supporting Information) and delithiation (Figure S36h, Supporting Information) only exhibit diffraction rings of (002), (100), (102), and (110) planes of the vertical graphene structure, suggesting that the formation of Li*_y_* Si (discharged to 0.005 V) and Si (charged to 3.0 V) is amorphous, which is in agreement with the results of Ex situ XRD (Figure 6e). Additionally, ex situ EIS illustrates that the resistance decreases gradually due to electrode activation during the first (de)lithiation process, indicating a stable and highly conductive electrode (Figure 6g and Figure S29b, Supporting Information). Upon different (de) lithiation stages, XPS spectra of C 1s contains C—C, C═O, and C═N peaks for all potential ranges, the peak of C═O increases due to the decomposition of electrolyte on the VGAs@Si@CNFs‐1 electrode (Figure 6h). As for F 1s characteristic spectrum (Figure 6i), the peaks centered at 685.8 and 688 eV can be ascribed to the formation of LiF and P‐F and exhibit high intensity for (de)lithiation states, which demonstrates a more stable SEI layer for enhanced cycling stability.^[^ ^66^, ^67^ ^]^ The postmortem examination demonstrates that the unique coating of VGAs growth on the CNFs and Si is beneficial for the stable (de) lithiation process.

### 2.4 Evaluations on the Practical Prospect

**Figure 7:**
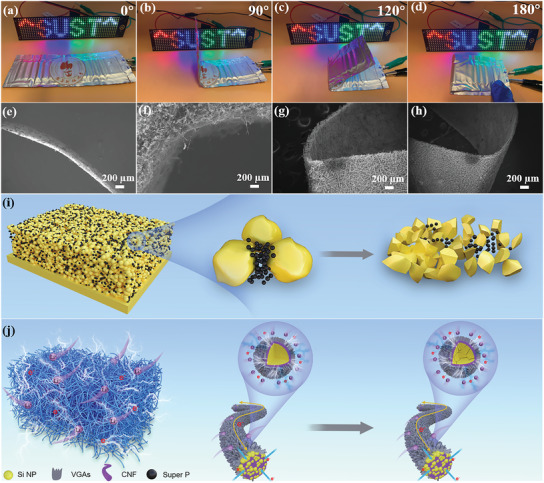
Practical applications of flexible pouch cells. a–d) Powering LED lighting by VGAs@Si@CNFs‐1/LCO full cell under various bending states and e–h) corresponding SEM images. Schematic illustrating the enhanced Li^+^ and electron transportation mechanisms between active materials and i) Si NPs anode, and j) the novel VGAs@Si@CNFs anode.

Considering the practical application and perusing high‐ energy‐density Li−Si cells, full cells were further assembled with commercial cathode materials (LiFePO_4_). The full cell VGAs@Si@CNFs‐1/LFP shows an obvious discharge platform from 3.2 to 3.4 V (Figure S37a, Supporting Information), acquiring a high nominal voltage of 3.3 V. The gravimetric energy density of VGAs@Si@CNFs‐1/LFP at 0.2 C can be calculated as 581.5 Wh kg^−1^. The VGAs@Si@CNFs‐1/LFP also delivers outstanding rate performance, demonstrating excellent fast charging performance and practicality (Figure S37b, Supporting Information). Moreover, we assembled pouch cells using VGAs@Si@CNFs‐1 and commercial LiCoO_2_ as the anode and cathode (VGAs@Si@CNFs‐1/LCO) (Figure S38a, Supporting Information). Ultrastable open‐circuit potential at 3.84 V is measured for VGAs@Si@CNFs‐1/LCO pouch cell under different bending states from 0 to 360 degrees (Figure S38b, Supporting Information). The flexible pouch cell is capable of powering light‐emitting diode (LED) during arbitrarily changing bending states, and continuously lighting the LED for more than 24 h (**Figure** **7**a–d and Video S3, Supporting Information). The VGAs@Si@CNFs‐1/LCO also possesses an outstanding cycling performance when bending, folding, and even curling the battery (Figure S38c, Supporting Information), demonstrating excellent flexibility of the VGAs@Si@CNFs‐1 electrode, evidenced by the SEM images (Figure 7e–h). Therefore, such free‐standing VGAs@Si@CNFs electrode could provide much higher energy density and excellent stability, especially outstanding flexibility.

Based on the above results, the excellent cycle stability and preferable rate capability of VGAs@Si@CNFs architecture may be attributed to the following mechanisms (Figure 7j). First of all, the high specific capacity is ascribed to ultra‐high Si content, which depends on the optimized electrospinning process. Second, the superior electrical conductivity and abundant conductive contacts originated from the ingenious 3D interconnected network promote the Li^+^ and electrons diffusion kinetics, rather than a single‐point‐contact mode for original Si NPs. Specifically, the CNFs network is regarded as building blocks to prevent the aggregation and pulverization of Si NPs during (de) lithiation. VGAs act as electrical roads and “bridges” among Si NPs and CNFs to improve the conductivity of the electrode and enlarge the contact area with electrolyte. Such a unique structure can efficiently alleviate the huge volume expansion/contraction of the electrode, maintaining the electrode integrity. Meanwhile, N, O co‐doped architecture can induce numerous extrinsic defects and active sites, which can further enhance the wettability of electrolyte and the lithium absorption properties.

## 3 Conclusion

In summary, we have successfully developed a synthesis strategy for the preparation of the free‐standing N, O co‐doped vertical graphene arrays grown on Si@CNFs structure (VGAs@Si@CNFs) as anode for LIBs. A series of VGAs@Si@CNFs films with adjustable morphology, size, and Si content were designed and fabricated. As a result, we demonstrate the superior electrochemical performance of the VGAs@Si@CNFs electrode with a ultra‐high Si content of 90.2%, including the high specific capacity and rate capability as well as outstanding cycling stability. In particular, the capacity of the VGAs@Si@CNFs‐1 electrode exhibits 3619.5 mAh g^−1^ at 0.05 A g^−1^ with persistent capacity retention of 2390.5 mAh g^−1^ after 100 cycles at 0.1 A g^−1^. Especially at 8 A g^−1^, it achieves a high capacity of 1093.1 mAh g^−1^ after 1500 cycles. The full cell and flexibility test further confirm the practicability and feasibility of free‐standing VGAs@Si@CNFs electrode, holding great potential in advanced energy storage and conversion devices for electric vehicles and hybrid electric vehicles and some portable flexible wearable products.

## Conflict of Interest

The authors declare no conflict of interest.

## Supporting information

Supporting Information

Supplemental Video 1

Supplemental Video 2

Supplemental Video 3

## Data Availability

Research data are not shared.
