# Supplementary material for: Nitrogen, Oxygen‐Codoped Vertical Graphene Arrays Coated 3D Flexible Carbon Nanofibers with High Silicon Content as an Ultrastable Anode for Superior Lithium Storage
Source: Adv Sci (Weinh). 2022 Jan 6;9(6):2104685. doi: 10.1002/advs.202104685 (PMC8867154; doi:10.1002/advs.202104685)
Supplement: Supplementary file 1 — Supporting Information [file ADVS-9-2104685-s003.pdf]

ADVANCED SCIENCE
Open Access

Supporting Information

for *Adv. Sci.*, DOI: 10.1002/advs.202104685

Nitrogen, Oxygen-codoped Vertical Graphene Arrays Coated 3D Flexible Carbon Nanofibers with High Silicon Content as an Ultrastable Anode for Superior Lithium Storage

Yongbiao Mu, Meisheng Han, Buke Wu, Yameng Wang, Zhenwei Li, Jiaxing Li, Zheng Li, Shuai Wang, Jiayu Wan, and Lin Zeng**

Supporting Information for

Nitrogen, Oxygen-codoped Vertical Graphene Arrays Coated 3D Flexible Carbon Nanofibers with High Silicon Content as an Ultrastable Anode for Superior Lithium Storage

Yongbiao Mu, Meisheng Han, Buke Wu, Yameng Wang, Zhenwei Li, Jiaxing Li, Zheng Li, Shuai Wang, Jiayu Wan, * and Lin Zeng*

*Corresponding author: wanjy@sustech.edu.cn (J. Wan); zengl3@sustech.edu.cn (L. Zeng)

1. Experimental Section

1.1 Materials

Silicon nanoparticles (Si NPs) with diameter (30-50 nm) were purchased from Shanghai Hanlane New Material Technology Co., Ltd. Polyacrylonitrile (PAN, MW=150000 g mol^{-1}), N, N-dimethylformamide (DMF) were purchased from Shanghai Macklin Biochemical Co., Ltd. Stainless steel mesh of 100-400 mesh were purchased from Shenzhen Xianglong Qirui Technology Co., Ltd. For coin-cell, the electrolyte was purchased from DoDoChem. Conductive carbon black (Super P), lithium iron phosphate ($LiFePO_4$, LFP), nickel cobalt lithium manganate ($LiCoO_2$, LCO) Li foil (0.45mm, 99.9%), Cu foil (9 μm, 99.99%), Al foil (20 μm, 99.99%), polyvinylidene fluoride (PVDF), sodium carboxymethyl cellulose (CMC), styrene-butadiene rubber (SBR), Celgard 2400 membrane, ethanol (C_2H_5OH), and n-methyl-2-pyrrolidone (NMP) were purchased from MTI Corporation. Ar (purity: 99.99%), NH_3 (99.999%) and H_2 (purity: 99.99%) were purchased from Shenzhen Xiangyuan Industrial Gas Co., Ltd.

1.2 Synthesis of Si@CNFs composites

The Si@CNFs composites were prepared through an electrospinning technique followed by series of heat treatment processes. Typically, PAN (1g) as polymer skeleton was firstly dissolved in DMF (10 mL) to obtain a 10 wt% PAN solution by vigorous stirring for 2 h, and a certain amount of Si NPs (0.2 g, 0.5 g, 1 g, 2 g) were then added to above solution and stirred for another 4 h. The obtained homogenous mixture was used as the spinning solution for electrospinning and transferred into 10 mL syringe

with a 15-gauge stainless steel needle, and electrospinning process was conducted at a positive potential of 25 kV and negative potential of 2 kV. During the electrospinning process, stainless steel mesh (30cm x 15cm) was applied as the grounded collecting substrate, with a distance of 10 cm and a feeding rate of 1.2-3 mL h^{-1}. The humidity and temperature of the electrospinning chamber were controlled at 45 ± 5 % and 25 ± 2 °C, respectively. The as-collected polymer nanofibers were dried in the oven at 80 °C for 6 h for removing excess solvents and moisture absorbed in the air. Then the as-prepared Si@PAN nanofibers were pre-oxidized at 260 °C for 4h in the ambient air to stabilize the nanofiber structure and subsequently carbonized in a tube furnace at 1000 °C for 4 h under Ar (100 mL min^{-1}) or NH_3 (50 mL min^{-1}) atmosphere with a heating rate of 5 °C min^{-1}. For comparison, the pure CNFs without Si nanoparticles were obtained under the same conditions of stabilization and carbonization. In the stabilization process, a graphite plate clamp was designed to optimize the entire thermal-treatment stage. The as-prepared Si@PAN nanofibers were pre-oxidized through three different stabilization strategies at 260 °C, including natural placed, uniaxial thermal-stretching and biaxial thermal-stretching. These three strategies are also used in the carbonization process to obtain samples with different mechanical properties.

1.3 Synthesis of VGAs@Si@CNFs composites

The VGAs@Si@CNFs composites were obtained through a thermal CVD process for growing VGAs on Si@CNFs. First, the as-prepared Si@CNFs and pure CNFs were cut into any size and placed in the furnace center. Second, the tube was purged with 200 mL min^{-1} Ar to remove air, and then the furnace was heated to 950 °C at a rate of 10 °C

min^{-1} under Ar (100 mL min^{-1}) at ambient pressure. Third, when the temperature reached 950 °C, the ethanol used as a carbon source was carried into the quartz furnace through Ar via our self-made equipment with 50 mL min^{-1} flow rate of Ar. The VGAs can be grown successfully between 1000 °C and 1300 °C based on our previous experience. Fourth, When the temperature reached 1000 °C at a rate of 5 °C min^{-1} under Ar/C_2H_5OH (100 mL min^{-1}), it was time to grow VGAs on Si@CNFs by introducing H_2 at a flow rate of 135 mL min^{-1}. At this stage, the Si@CNFs composites were held for 3-6 h for vertical graphene growth. After growth, the furnace was naturally cooled to room temperature. The VGAs@Si@CNFs composites were obtained finally.

1.4 Materials Characterizations

The morphology of the VGAs@Si@CNFs was characterized by field emission scanning electron microscopy (SEM, JEOL-6700F). Transmission electron microscopy (TEM), energy-dispersive X-ray analysis (EDX) and elemental mapping were performed using a Talos instrument with an acceleration voltage of 300 kV. X-ray diffraction (XRD, Bruker Advance D8, Ultima IV with D/teX Ultra with Cu-Kα radiation) was employed to characterize the crystalline structures of samples with a scanning rate of 5° min^{-1}. X-ray photoelectron spectra (XPS, Escalab 250Xi) were acquired on a Thermo SCIENTIFIC ESCALAB 250Xi with Al Kα (hυ = 1486.8 eV) as the excitation source. Raman spectra were performed on a HORIBA LabRAM HR Evolution using a 532 nm laser as the excitation source. A typical four-probe method (RTS-8) was used to measure the conductivity of the as-prepared nanofiber composites materials. The content of Si NPs in the electrodes was measured by thermogravimetric analysis (TGA, 209 F1), from room temperature to 1000 °C under air with a heating rate of 10 °C min^{-1}. The calculation of Si content based on the equation: [1,2]

$$\boldsymbol{W_{Si/VGAs@Si@CNFs} = W_{Si} \times X_{Si} + (-W_C) \times (1 - X_{Si})}$$

Where $W_{Si/VGAs@Si@CNFs}$, W_{Si} and W_C correspond to the residual weight percentage

of Si/VGAs@Si@CNFs electrodes, original Si NPs and C, and X_{Si} represent the required content of Si. The N_2 adsorption–desorption isotherms and pore size distribution of the samples were achieved by a fully automatic specific surface area and pore size tester (ASAP2460).

The resistance of the powders was determined by a resistivity tester (ST2722.SZ). During the testing process, the powder with a volume of 1.5 mL was put into the test instrument, which was pressurized gradually. Finally, a small circular plate with a diameter of 10 mm and a controllable thickness was obtained. The electrical conductivity of the films was measured by four-probe instrument. The film is cut into small discs with a diameter of 10mm. The thickness of the film is controlled under the same conditions (100-200 μm) , the temperature and humidity are 45 ± 5% and 25 ± 2°, respectively.

1.5 Electrochemical Measurements

For half cells, the as-prepared free-standing CNFs, Si@CNFs, VGAs@CNFs, and VGAs@Si@CNFs films were cut into small pieces with diameter of 10 or 12 mm and then directly used as the working electrodes (The total mass of VGAs@Si@CFs was used to calculate the capacity during the electrochemical test) in CR2032 coin cells. For Si NPs electrode, working electrodes were made by applying slurries consisting of active materials, carbon black, SBR, and CMC at a mass ratio of 95:1.5:1.5:2 on Cu foil followed by drying at 85 °C for 12 h under vacuum. The weight of various electrodes is 0.8-1.5 mg cm^{-2}. The cells were assembled in an Ar-filled glove box (H_2O and O_2 contents < 0.01 ppm). Lithium foil and Celgard 2400 separator were applied as the counter electrode and separator membrane, respectively. 1 M $LiPF_6$ dissolved in ethylene carbonate (EC)+ dimethyl carbonate (DEC) + dimethyl carbonate (DMC)

(1:1:1 by volume) + 5% fluoroethylene carbonate (FEC) was utilized as the electrolyte. The electrochemical performance was tested using a Neware battery test system (Shenzhen, China) at a voltage interval of 0.005-3.0 V (versus Li/Li^+) with different current densities at 30 °C. Capacity was calculated on the basis of the total mass of the entire VGAs@Si@CNFs. The cyclic voltammetry (CV) curves at various scan rates and the electrochemical impedance spectroscopy (EIS) from 10^5 to 10^{-2} Hz with an amplitude of 5 mV were obtained on an electrochemical workstation (CHI 760D, Shanghai CH Instruments Co., China).

For full cells, the cathode electrodes were obtained by coating a mixture of LFP, carbon black, and PVDF at a mass ratio of 94:3:3 on Al foil. The cycling curves were measured at 0.1 and 1 C and rate curves were tested at 0.2-5 C in the voltage window of 2.5-3.8 V. In full cells, the capacity was calculated based on the active mass of LFP. All cells were tested by battery test system at 30 °C.

For pouch cells, the cathode electrodes were obtained by coating a mixture of LCO, carbon nanotubes, and PVDF at a mass ratio of 94.5:2.5:3 on Al foil. The loading of LCO cathode is about 8.3 mg cm^{-2}, and the VGAs@Si@CNFs and LCO electrodes are cut into 11 cm x 2.5 cm. The cycling curves were measured at 0.1 C in the voltage window of 3.0-4.35 V. In full cells, the capacity was calculated based on the active mass of LCO.

The gravimetric/volumetric energy density of the full cell are calculated based on the following equation：

$$Gravimetric\ energy\ density\ (Wh\ kg^{-1}) = \left(\frac{Cc \times V}{(m_{cathode} + m_{anode})}\right)$$

where C_c is cell capacity (2.318 mAh), V is nominal voltage (3.3 V), $m_{cathode}$ and m_{anode} are active mass of cathode/anode.

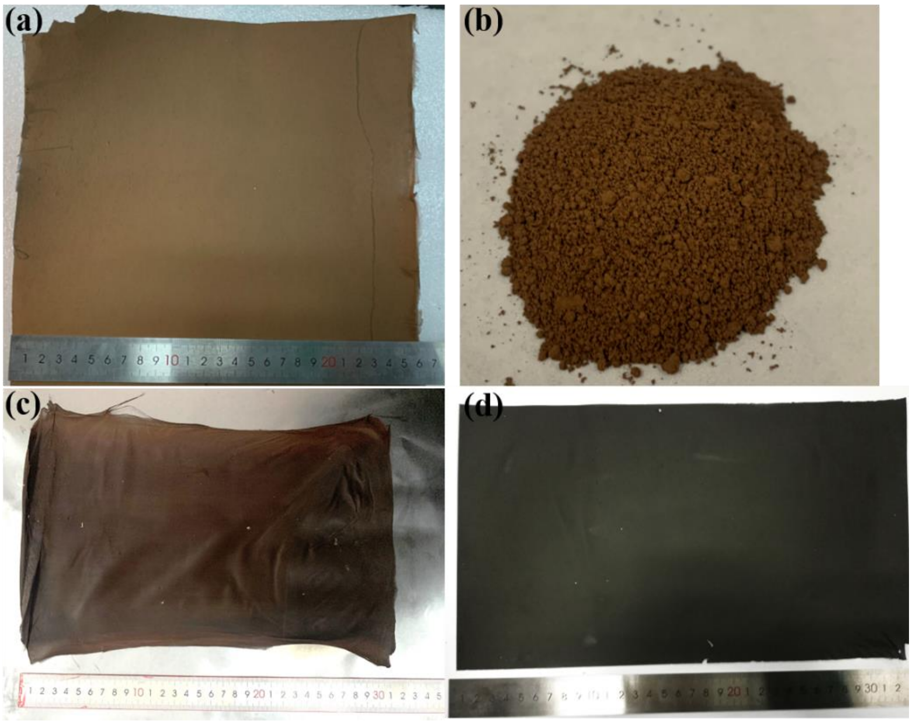

Figure S1. Photographs of (a) the Si@PAN fibers after electrospinning, (b) the Si NPs raw material, (c) the Si@PAN fibers after pre-oxidation in air atmosphere and (d) the Si@CNFs after carbonization.

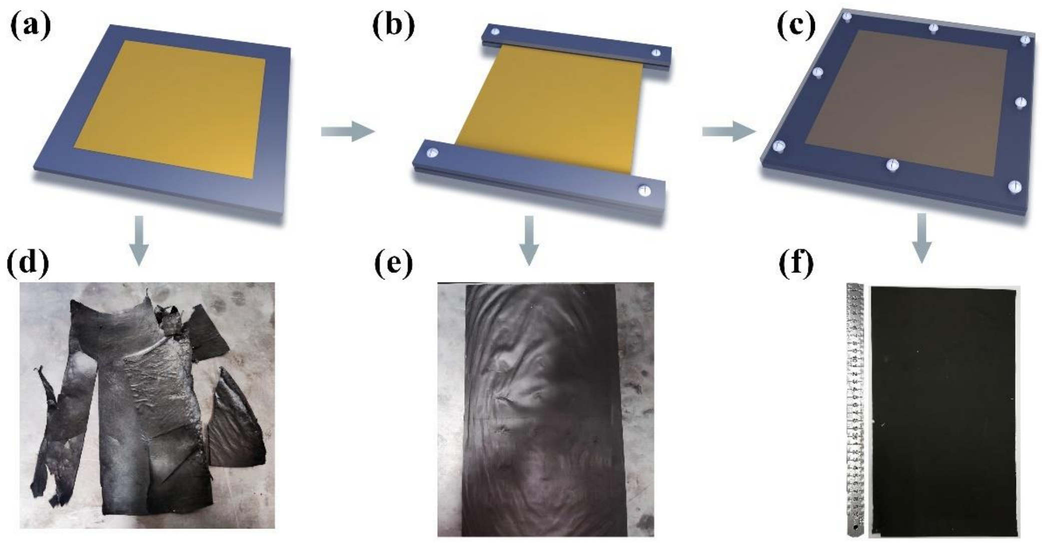

Figure S2. Schematics illustrating the thermal-treatment strategies of Si@CNFs electrodes. Si@PAN precursor fibers and Si@CNFs electrodes (a, d) naturally placed on the graphite plate, (b, e) clamped both ends with graphite plates and hung; (c, f) clamped with two layers of graphite plate.

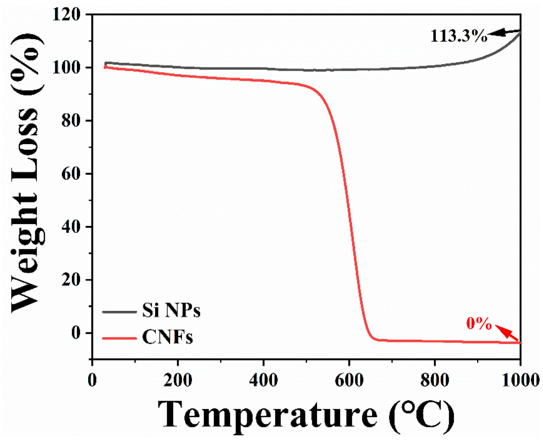

Figure S3. Thermogravimetric curves of the original Si NPs and the carbon nanofibers in air atmosphere.

Table S1. Weight distribution of Si in various VGAs@Si@CNFs electrodes.

Electrodes	W_{Si}(%)	W_C(%)	$W_{Si/VGAs@Si@CNFs}$(%)	X_{Si}(%)
VGAs@Si@CNFs-1	101.473	0	91.91	90.58
VGAs@Si@CNFs-2	101.473	0	72.9	71.84
VGAs@Si@CNFs-3	101.473	0	54.57	53.78
VGAs@Si@CNFs-4	101.473	0	18.15	17.89

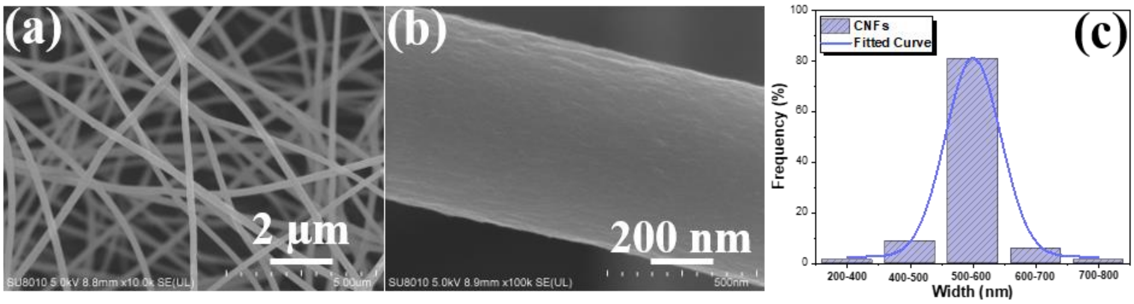

Figure S4. (a, b) SEM images and (c) width distributions of the CNFs.

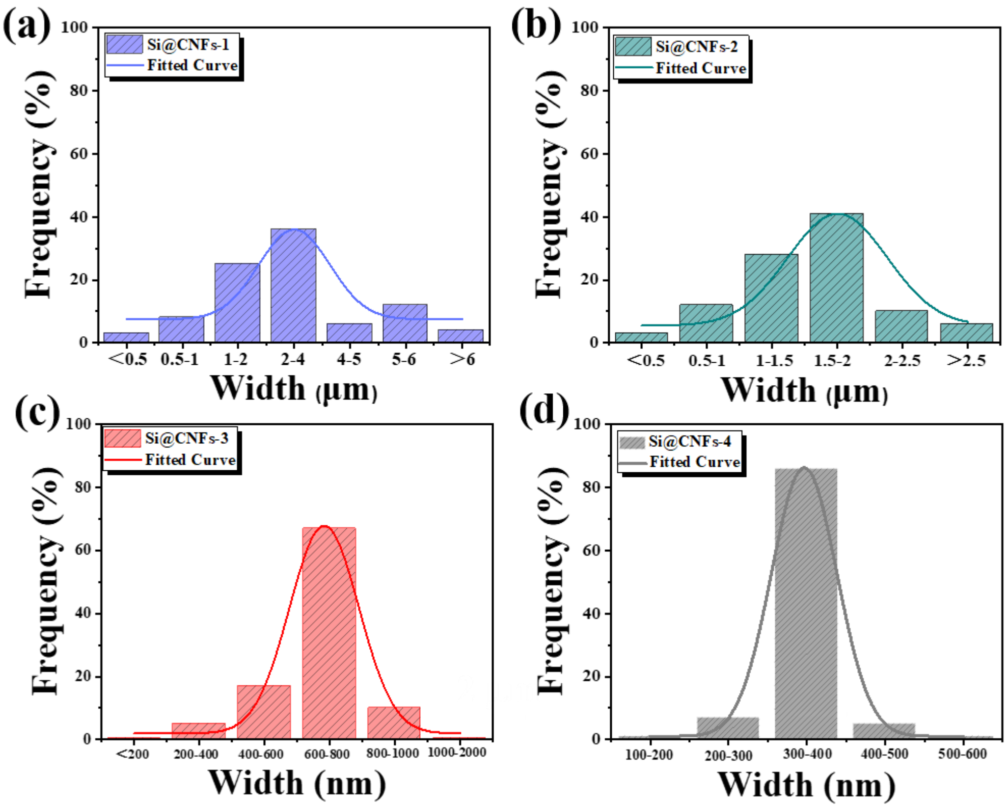

Figure S5. Width distributions of the (a) Si@CNFs-1, (b) Si@CNFs-2, (c) Si@CNFs-3, (d) Si@CNFs-4.

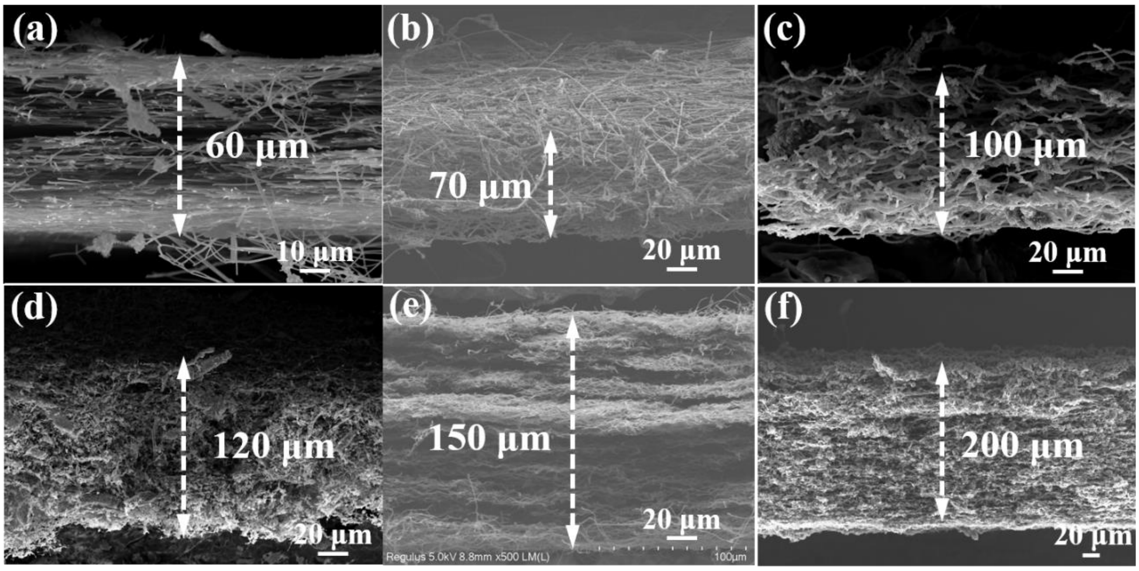

Figure S6. The cross-section SEM image of Si@CNFs-1 by adjusting the time of electrospinning in the range of (a) 2 h, (b) 2.5 h, (c) 3 h, (d) 4 h, (e) 5 h, and (f) 6 h.

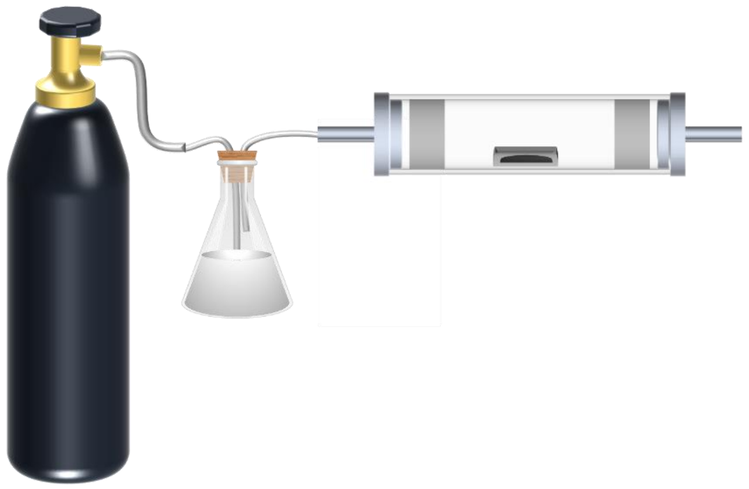

Figure S7. A self-made equipment for introducing ethanol vapor as the carbon source during CVD process.

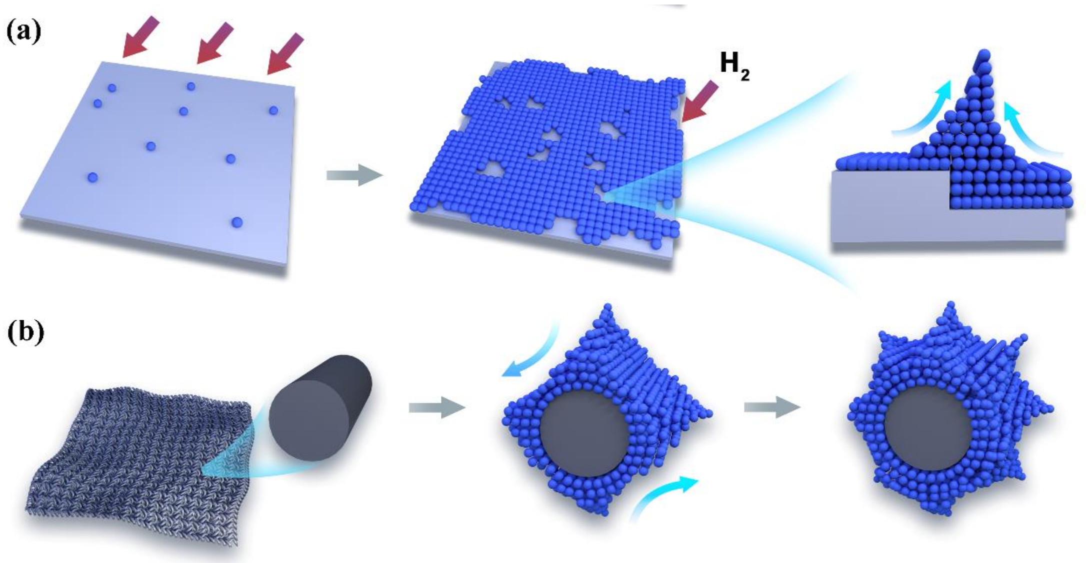

Figure S8. (a) Schematics illustrating of vertical graphene nanosheets arrays growth on a flat substrate; (b) schematics illustrating of vertical graphene nanosheets arrays growth on a high curvature Si@CNFs.

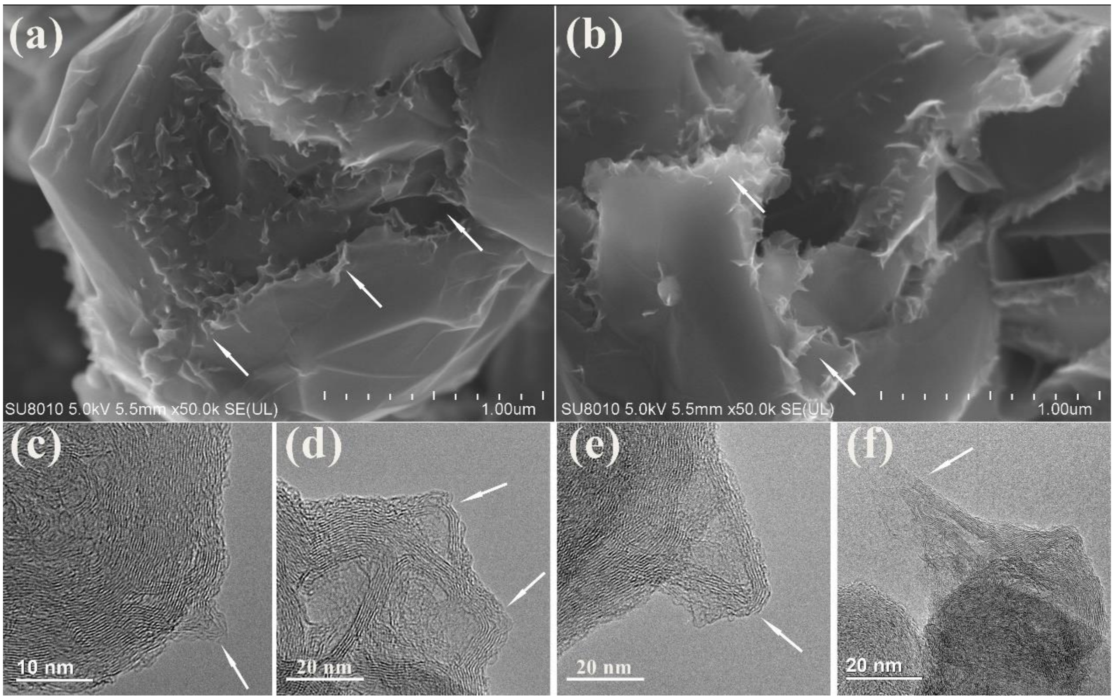

Figure S9. (a, b) SEM images of vertical graphene nucleation at steps and defects; (c-d) TEM images of vertical graphene nanosheets of nucleation and growth on a highly

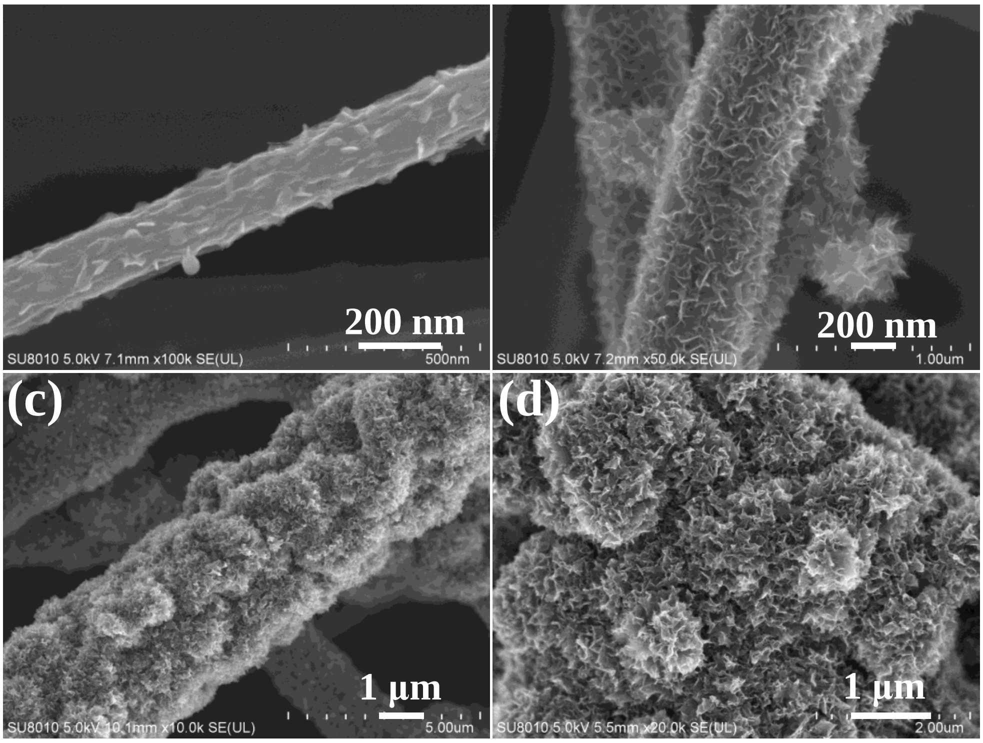

Figure S10. SEM images of VGAs@Si@CNFs for (a) 0.5 h, (b) 1 h, (c) 2 h, (d) 3 h growth time.

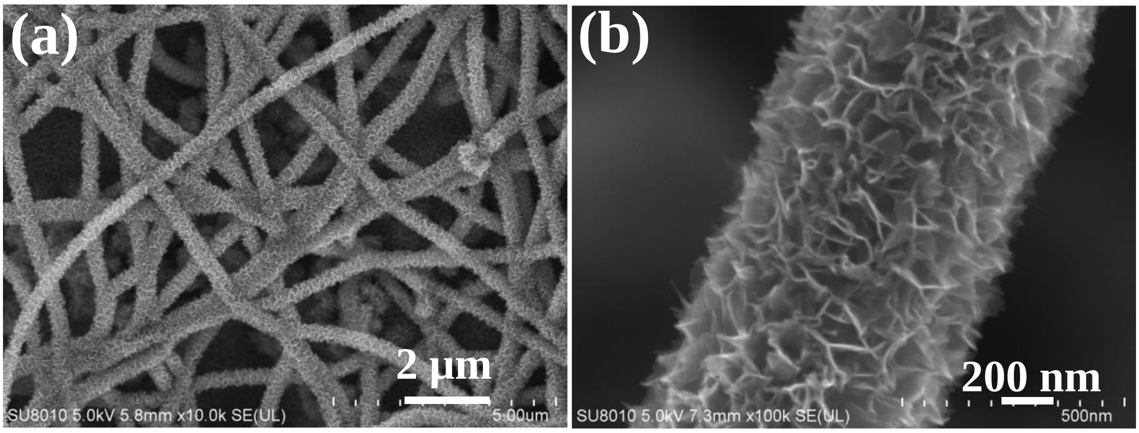

Figure S11. SEM images of VGAs@CNFs for 6 h growth time.

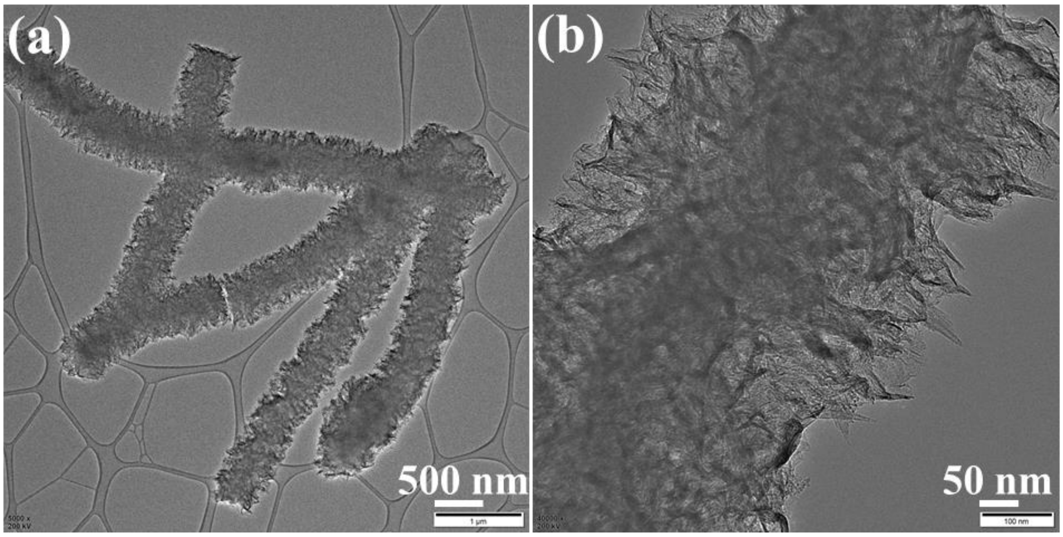

Figure S12. TEM images of original CNFs for 6 h growth time.

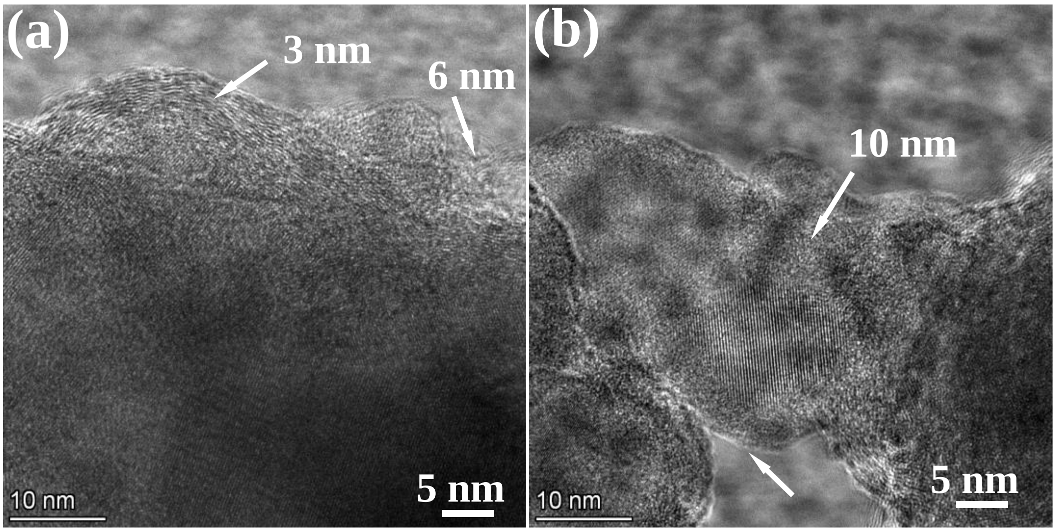

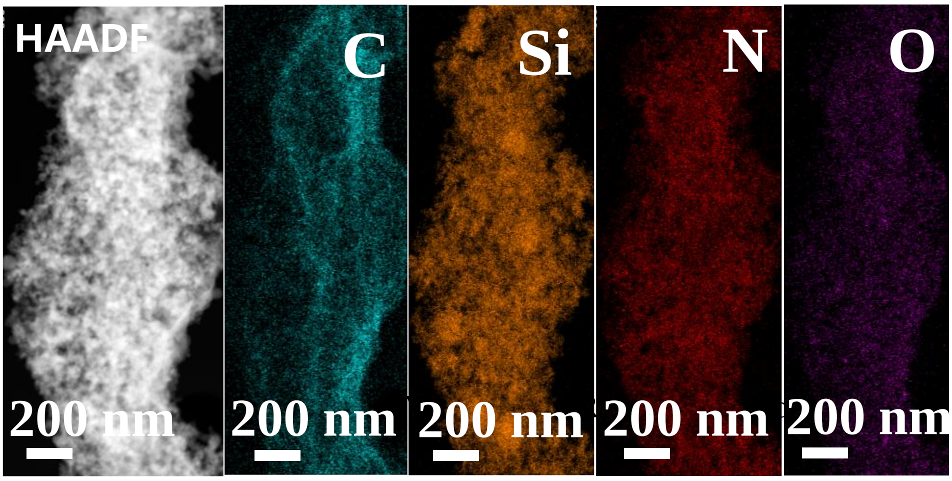

Figure S14. The EDS for separate elementary mappings of Si@CNFs.

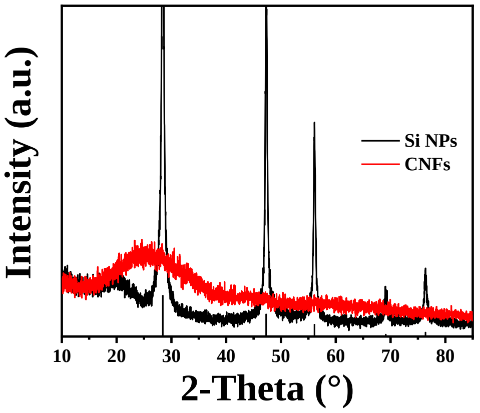

Figure S15. The XRD patterns of CNFs and Si NPs.

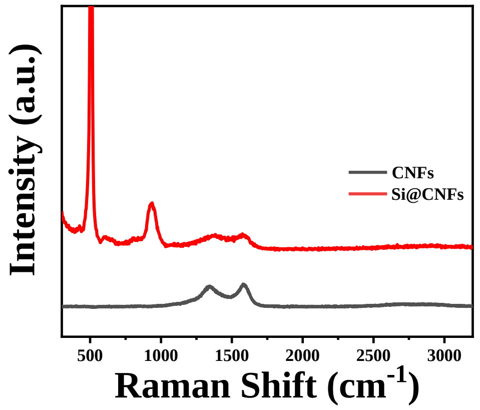

Figure S16. The Raman spectroscopy of CNFs and Si@CNFs.

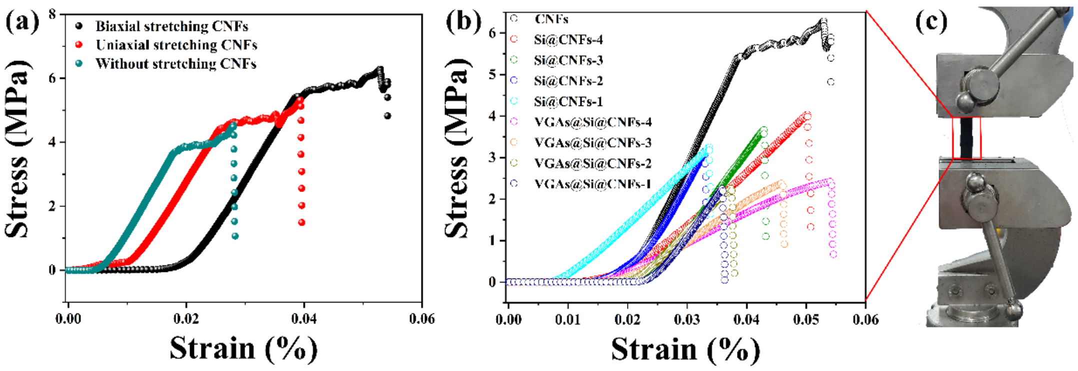

Figure S17. (a) The tensile stress-strain curves of the CNFs films under three pre-

oxidation modes and (b) various free-standing electrodes; (c) optical picture of test equipment.

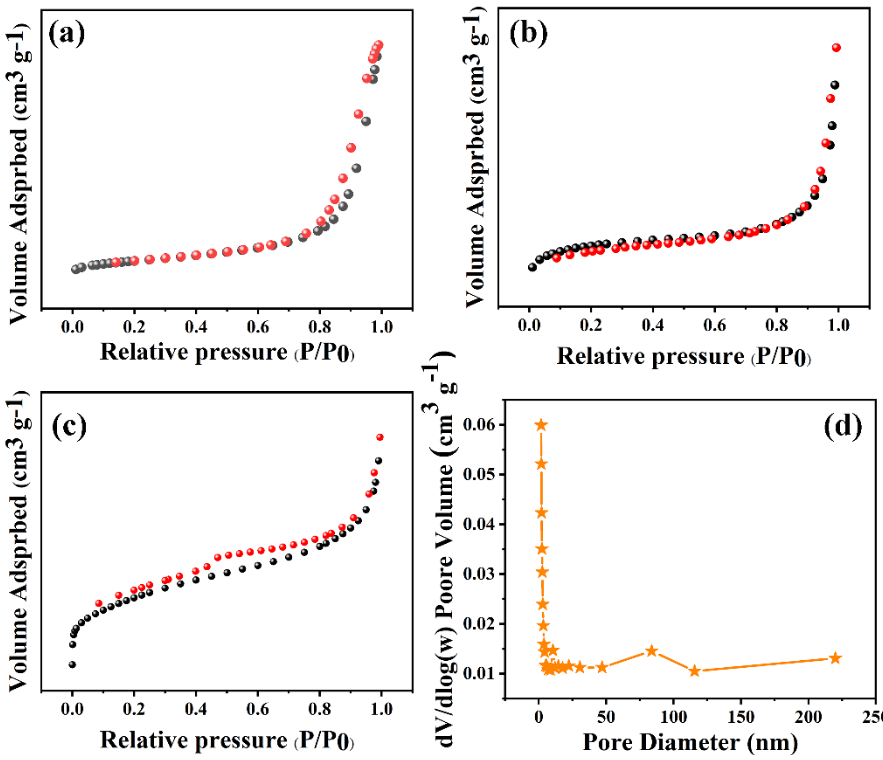

Figure S18. Nitrogen adsorption/desorption isotherms of (a) VGAs@Si@CNFs-1, (b) CNFs and (c) Si@CNFs and (d) pore size distribution of VGAs@Si@CNFs-1.

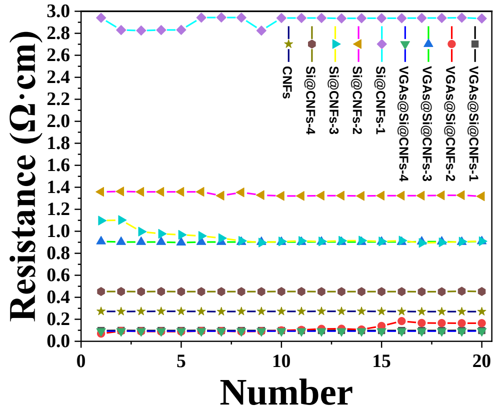

Figure S19. The resistance of the various electrodes measured using the four probes method.

Table S2. The weight contents of every element in various samples.

Samples	C contents (%)	Si contents (%)	N contents (%)	O contents (%)
VGAs@Si@CNFs-1	57.30	38.36	3.17	1.17
VGAs@Si@CNFs-2	67.68	27.30	3.71	1.31
VGAs@Si@CNFs-3	78.48	12.14	4.68	4.70
VGAs@Si@CNFs-4	84.90	7.40	4.73	2.97

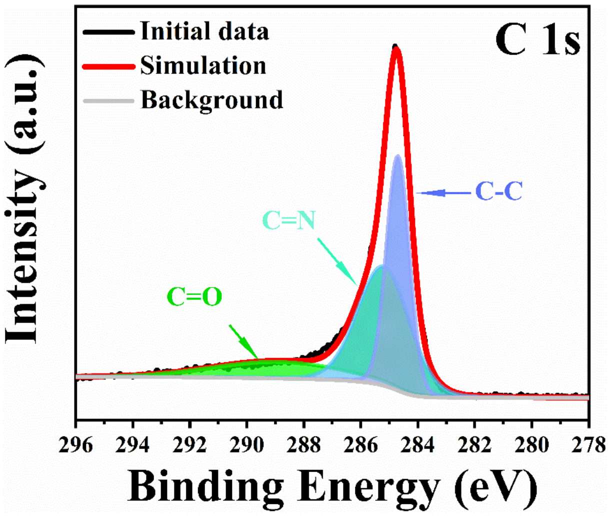

Figure S20. The XPS spectra of high-resolution C 1s.

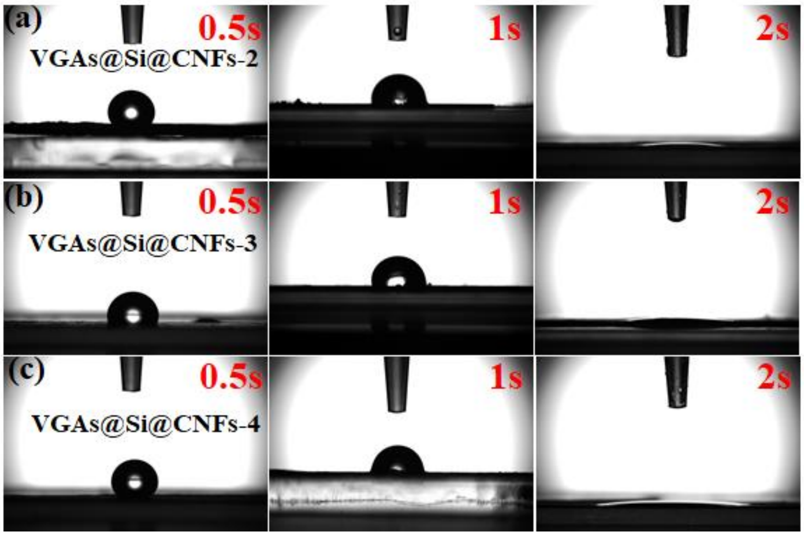

Figure S21. The contact angle (CA) measurements of (a) VGAs@Si@CNFs-2, (b) VGAs@Si@CNFs-3 and (c) VGAs@Si@CNFs-4.

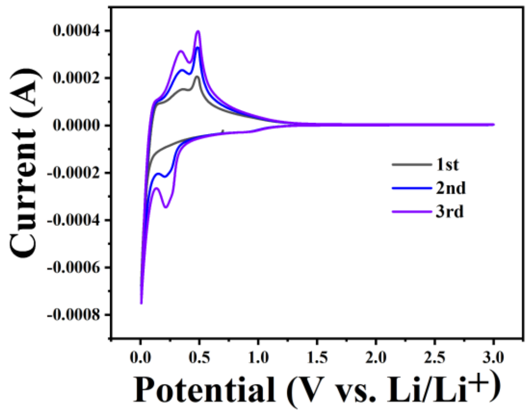

Figure S22. The CV curves of the Si@CNFs-1.

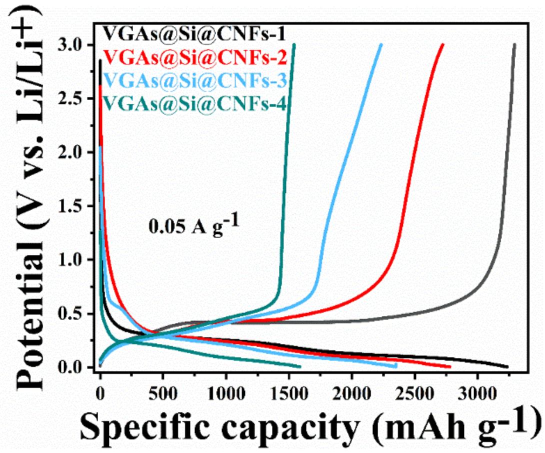

Figure S23. The galvanostatic charge/discharge profiles for VGAs@Si@CNFs-X (X=1, 2, 3, 4) at 0.05 A g^{-1}.

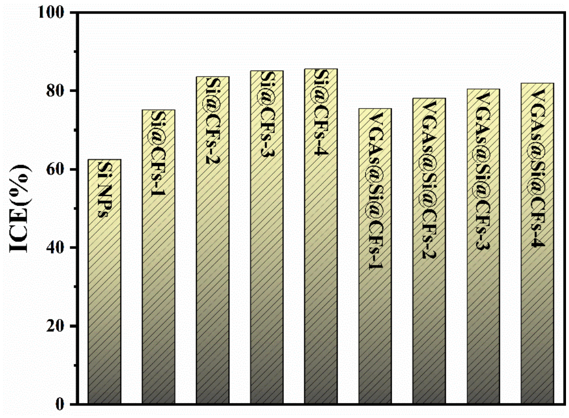

Figure S24. The initial coulombic efficiency (ICE) for various electrodes.

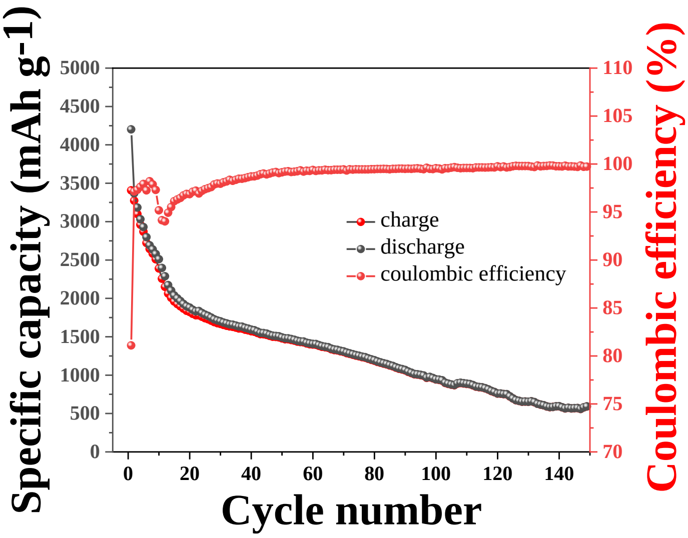

Figure S25. The cycle performance of Si NPs at 0.1A g^{-1}.

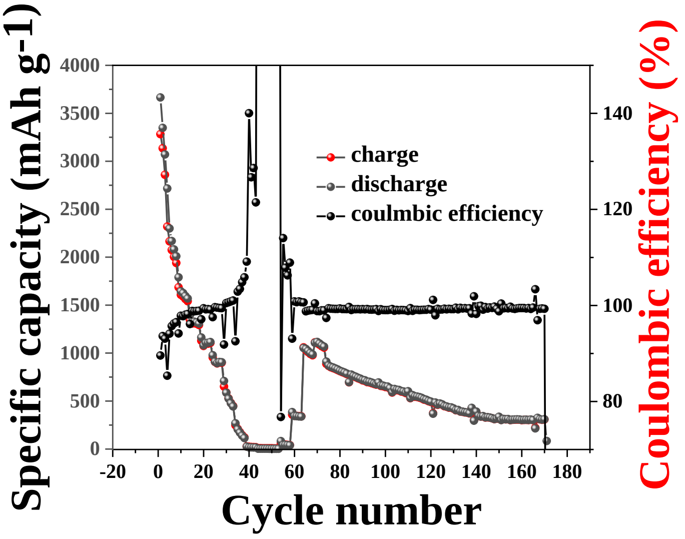

Figure S26. The rate performance of Si NPs.

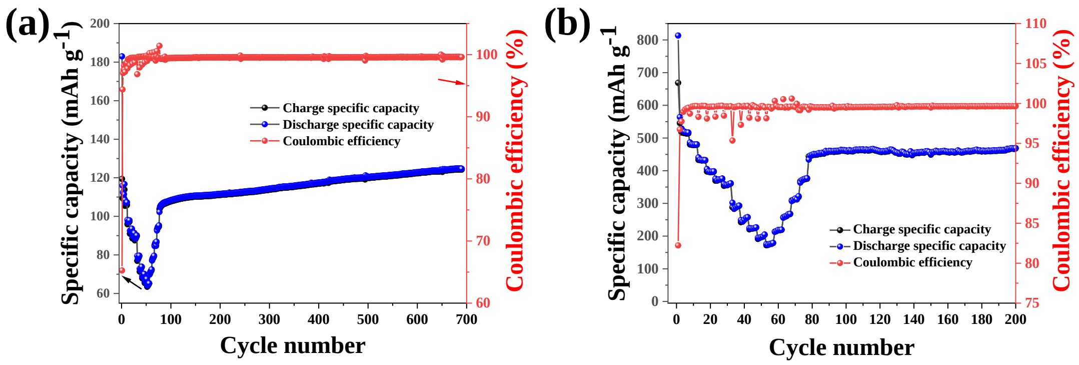

Figure S27. The rate and cycling performance of CNFs and VGAs@CNFs.

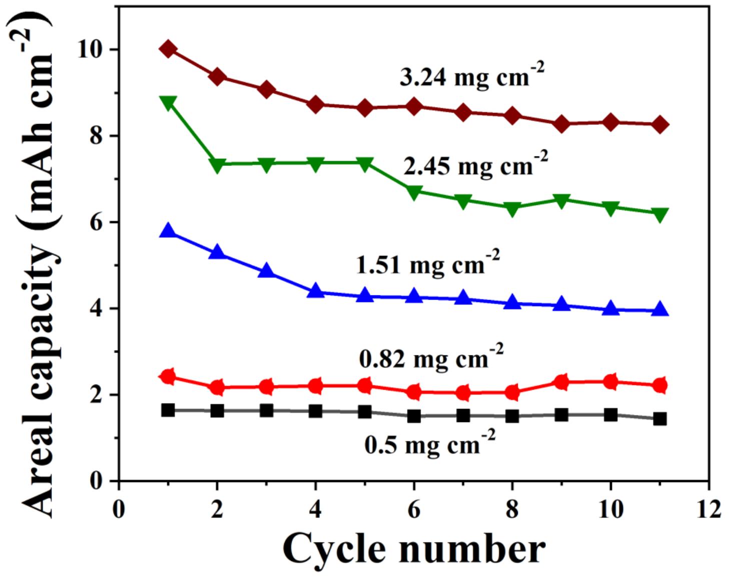

Figure S28. The areal capacities of the VGAs@Si@CNFs at a slow rate of 0.1 A g^{-1} with different mass loadings.

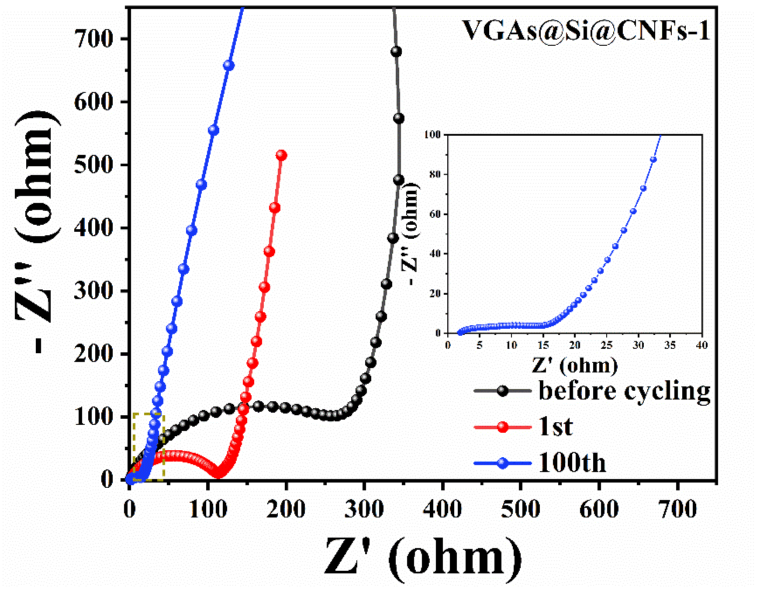

Figure S29. The electrochemical impedance spectra (EIS) of VGAs@Si@CNFs-1 electrode before and after cycling.

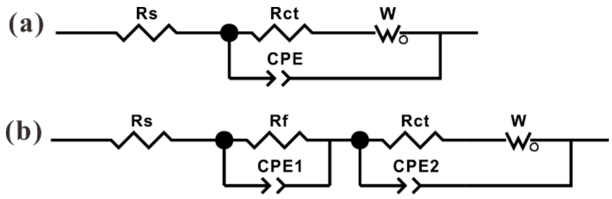

Figure S30. Equivalent circuit models used for EIS spectra: (a) before cycling and (b) cycling at different charge/discharge stages.

Table S3. The values of R_s, R_f, and R_{ct} of VGAs@Si@CNFs-1 electrode before cycling and cycling.

Samples	R_s (Ω)	R_f (Ω)	R_{ct} (Ω)
VGAs@Si@CNFs-1, before cycling	1.548	0	358.6
VGAs@Si@CNFs-1, after 1th cycling	1.852	12.2	112.9
VGAs@Si@CNFs-1, after 100th cycling	1.92	8.6	17.5

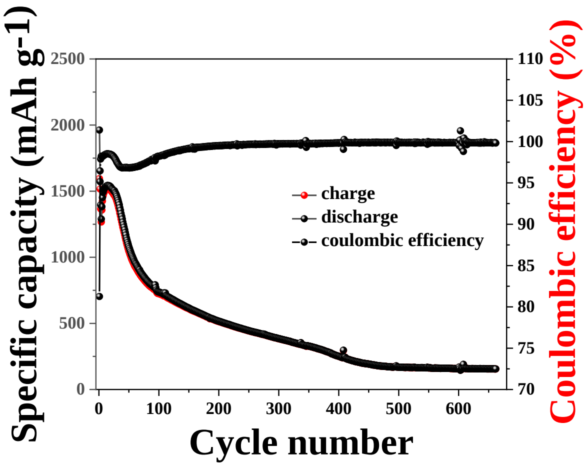

Figure S31. The cycle performance of Si NPs at 1A g^{-1}.

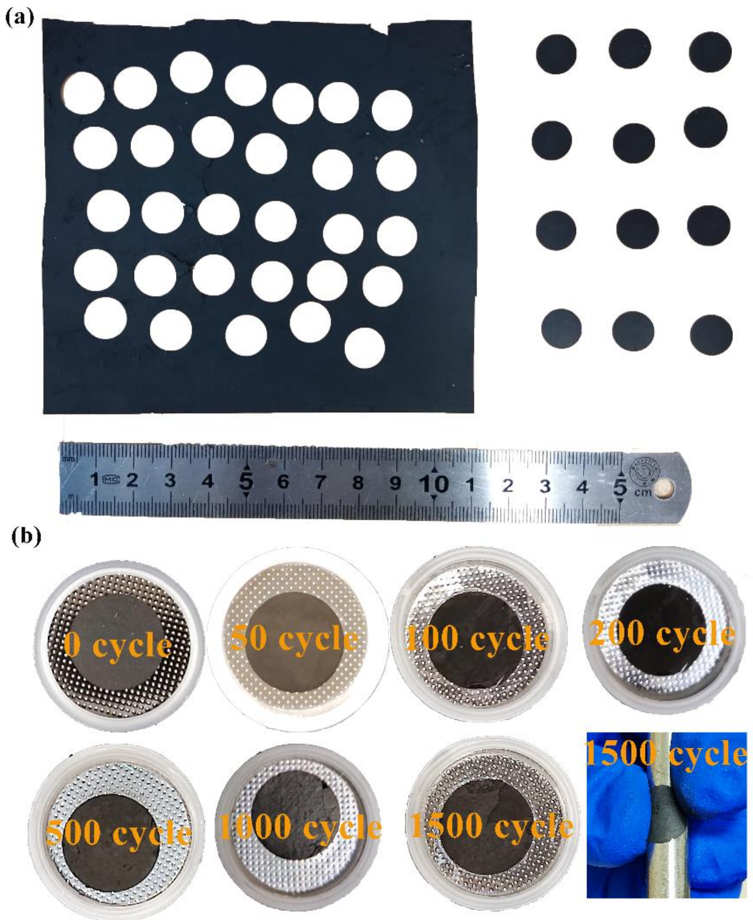

Figure S32. Optical photograph of (a) VGAs@Si@CNFs electrodes with diameter of 11 mm and (b) VGAs@Si@CNFs electrodes after different cycling number.

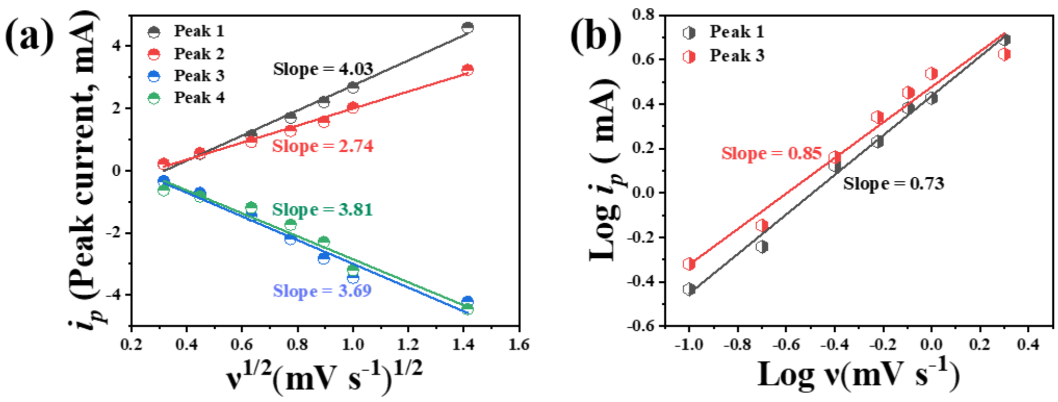

Figure S33. (a) The relationship between the square root of v and peak current, (b) Log (current, mA) versus Log (scan rate, mV s^{-1}) plots at specific peak currents.

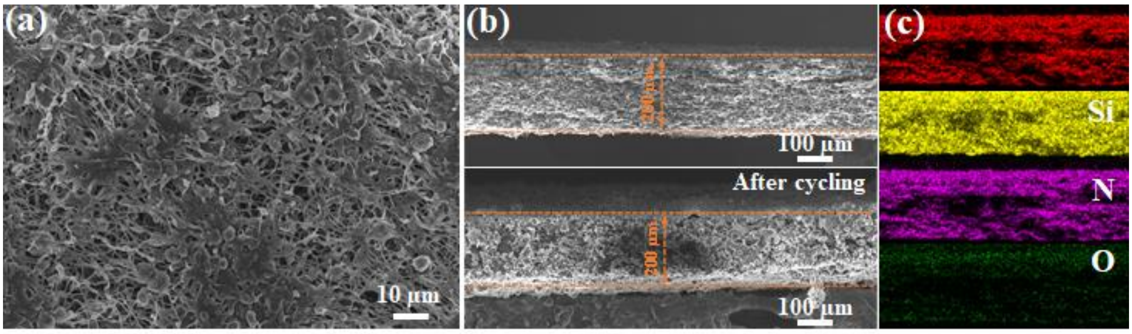

Figure S34. (a) Top-side SEM images after 10 cycles, (b) cross-sectional SEM images before and after 100 cycles, (b) corresponding to the EDX mapping data.

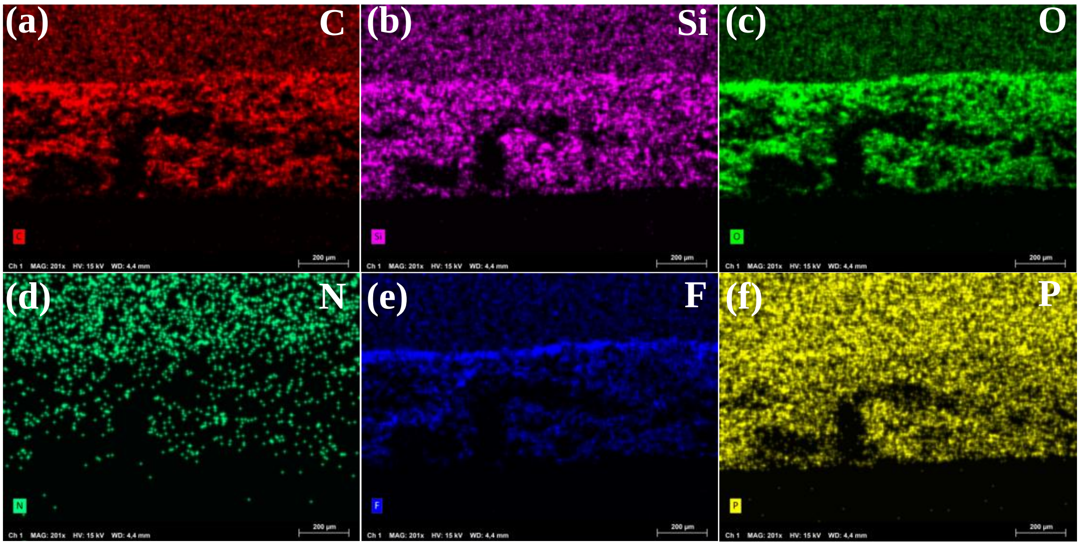

Figure S35. The VGAs@Si@CNFs-1 electrode cross-sectional EDS mapping data of (a) C, (b) Si, (c) O, (d) N, (e) F, and (f) P elements after cycling.

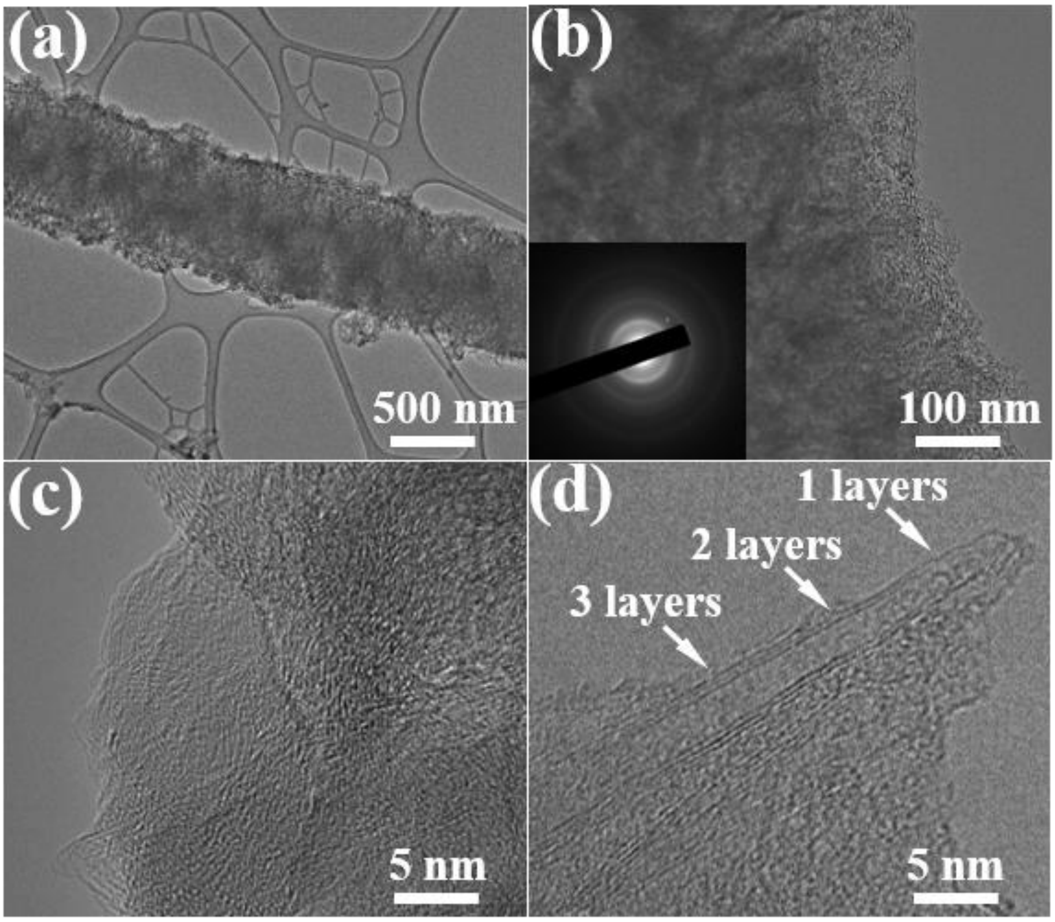

Figure S36. The ex-situ TEM images after cycling of (a) VGAs@Si@CNFs electrode, and (b, c) HRTEM images. (d) the top of vertical graphene nanosheets after cycling.

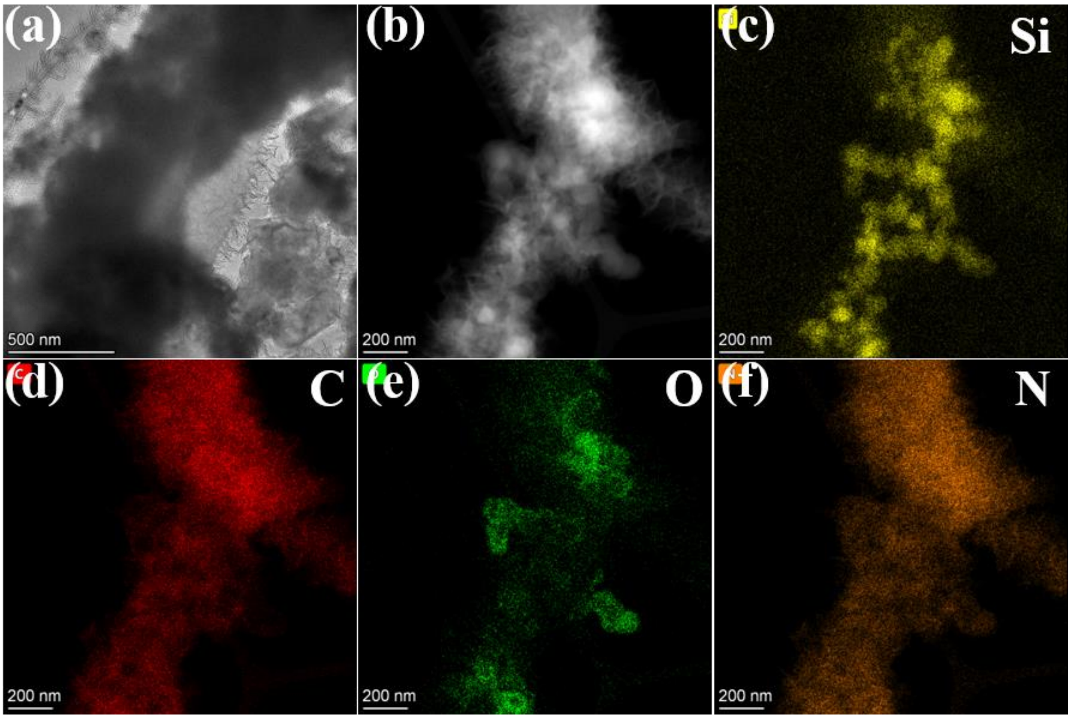

Figure S37. The TEM images (a) and EDS mapping data of (c) Si, (d) C, (e) O, and (f) N elements after cycling for VGAs@Si@CNFs electrode.

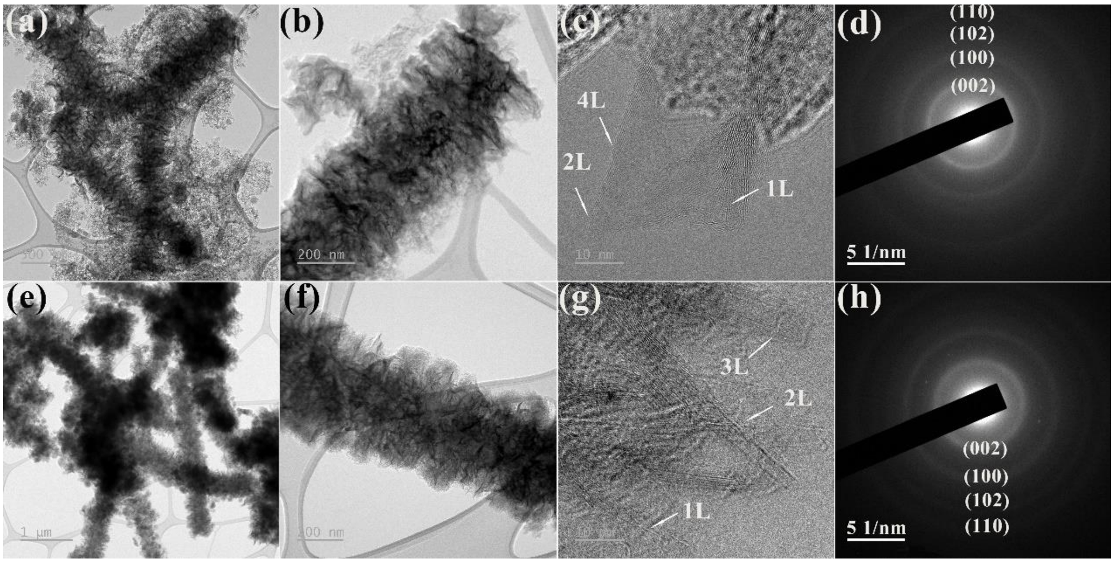

Figure S38. TEM images of VGAs@Si@CNFs-1 electrode with (a, b) complete lithiation and (e-g) delithiation; (c) HRTEM image of the top structure of vertical graphene nanosheets; the selected area electron diffraction (SAED) patterns at different states of (d) the completely lithiated and (h) delithiated.

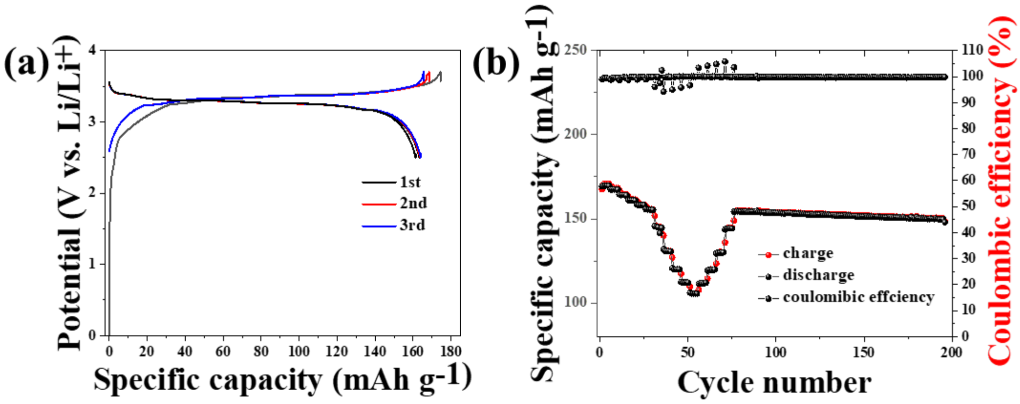

Figure S39. The full cell performance of VGAs@Si@CNFs-1/LFP for (a) charge/discharge curves and (b) rate and cycling data.

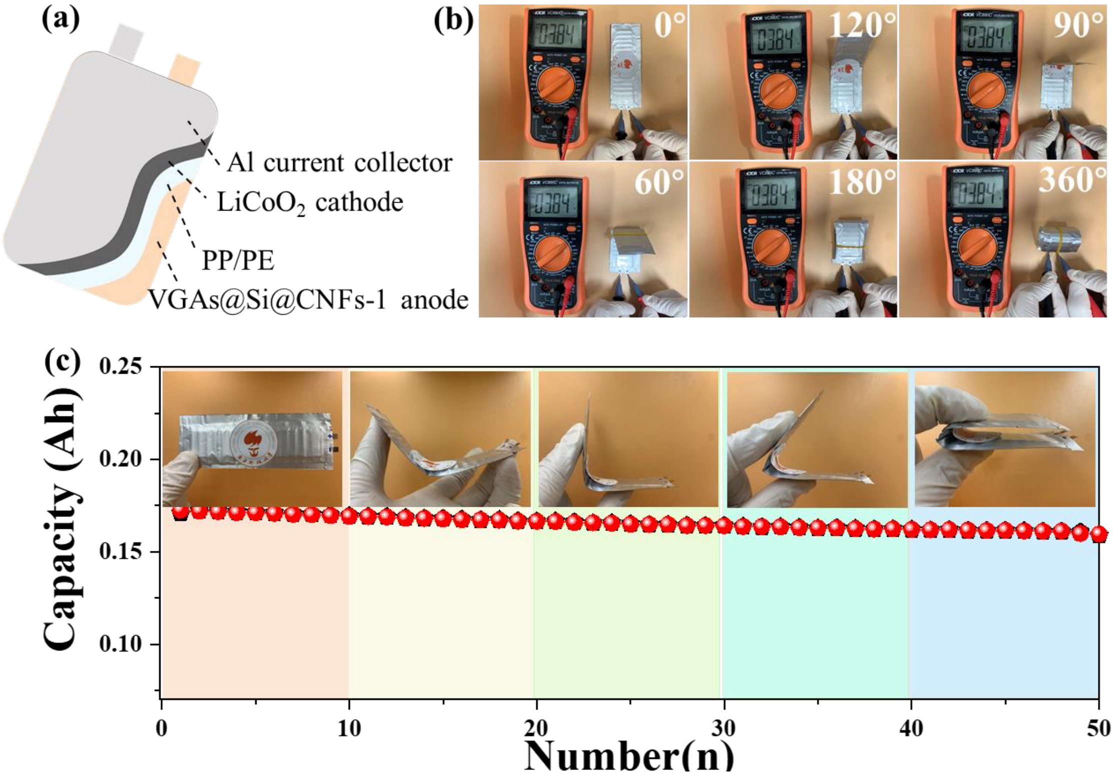

Figure S40. (a) Composition diagram of pouch cell, (b) open-circuit voltage and (c) cyclic stability tests under different bending states.

Table S4. A summary of performances of different Si-based anodes in comparision with the results in this work.

Description	Si contents (wt%) in active materials	Reversible capacity (mAh g –1)	Capacity retention(%)	Reference
**VGAs@Si@CNFs**	**90.58**	**0.05 A g^{-1} 3619.5**		**This work**
		**0.1 A g^{-1} 3219.5**	**100 cycles 66.5%**	
		**1.0 A g^{-1} 2183.8**		
		**8 A g^{-1} 1093.8**	**1500 cycles 94.5%**	
		**10 A g^{-1} 1059.2**		
	**71.84**	**0.1 A g^{-1} 2968.2.5**	**100 cycles 70.1%**	
		**8 A g^{-1} 1007.4**	**1500 cycles 95.2%**	
	**53.78**	**0.1 A g^{-1} 2115.7**	**100 cycles 73.8%**	
		**8 A g^{-1} 976.9**	**1500 cycles 96.3%**	
	**17.89**	**0.1 A g^{-1} 1457.6**	**100 cycles 93.6%**	
		**8 A g^{-1} 523.4**	**1500 cycles 96.9%**	
SiO@vG		0.16 A g^{-1} 1600		*Nano Lett.*, ***2017***, *17, 3681.*
		0.32 A g^{-1} 1000	100 cycles 93%	
Graphene cage @SiMP		0.21 A g^{-1} 3300		*Nature Energy,* ***2016***, *1, 15029.*
		2.1 A g^{-1} 1400	300 cycles 85%	
Si@CNT/C-microscroll	92.2	0.2 A g^{-1} 3254		*Energy Environ. Sci.,* ***2020***, *13, 848.*
	84.5	0.2 A g^{-1} 2704	300 cycles 76%	
Si@void@C Nanofibers	58	0.5A g^{-1} 1045.2	100 cycles 88%	*ACS Nano,* ***2018***, *12, 4835.*

		5 A g^{-1} 400		
3D FSiGCNFs	47	0.7A g^{-1} 2002	1050 cycles 99.88%	*ACS Nano*, ***2016***, *10, 8243.*
		1.4A g^{-1} 1201	2800 cycles 99%	
VG@SiOx /NC	84.4	0.1A g^{-1}1229.2	100 cycles 92.9%	*Journal of Mater. Chem. A*, ***2020***, *8, 3822.*
		2A g^{-1} 766.8	500 cycles 84.2%	
		20A g^{-1} 265.5		
3D Si/C Fiber Paper	72	0.2A g^{-1} 1600	100 cycles 73% 600 cycles 52.5%	*Adv. Energy Mater.*, ***2015***, *5, 1400753.*
Nanocrystal-FeSi-embedded Si/SiOx		0.05A g^{-1} 931.3		*Energy Storage Mater.*, ***2017***, *8, 119.*
		1A g^{-1} 500	1000 cycles 86%	
Si/C-ZIF-8/CNFs	54.5	0.2A g^{-1} 945.5	150 cycles 64%	*J. Energy Chem.*, ***2020***, *54, 727.*
		0.5 A g^{-1} 538.6	500 cycles 68%	
		0.5 A g^{-1} 3420.8		
GSiWh	25	0.2 A g^{-1}804	200 cycles 74%	*Carbon*, ***2019***, *148, 36.*
Si/CNTs	45.34	0.2 A g^{-1}1460.1	100 cycles 87.4%	*Energy Storage Mater.*, ***2019***, *17, 93.*
		1 A g^{-1}1275.5	400 cycles 75.7%	
Hollow core-shell structured silicon@carbon		0.2 A g^{-1}1995.2	100 cycles 51.2%	*J. Power Sources*, ***2017***, *342, 467.*
		3.2 A g^{-1}90		
Sandwich-like CNTs/Si/C nanotubes	70	0.5 A g^{-1}1508.5	1000 cycles ＞ 100%	*J. Mater. Chem. A*, ***2018***, *6, 14797.*
		1 A g^{-1}1216.6	1000 cycles ＞ 100%	
		2 A g^{-1}932.2	1000 cycles ＞ 100%	
Si@SiC@C	92.6	0.5 A g^{-1}	550 cycles 52.5%	*Nano Lett.*, ***2019***, *19, 5124.*
		1 A g^{-1}1897.7	800 cycles51.6%	
ZnO-Si@C-PCNFs	29.7	0.2 A g^{-1} 995	50 cycles 97.5%	*Small*, ***2019***, *15, 1900436.*
		0.8 A g^{-1}1050	1000 cycles ＞	

			100%	
CNT-wedged micro porous Si		0.4 A g^{-1} 2213.4	100 cycles 91.7%	*J. Power Sources*, ***2017***, *348, 302.*
SiOx/C	30	0.13 A g^{-1} 645	500 cycles 90%	*Adv. Funct. Mater.*, ***2018***, *28, 1705235.*
Graphdiyne/Si/Cu	30-40	0.2 A g^{-1} 4122		*Adv. Mater.*, ***2018***, *30, 1801459.*
		10 A g^{-1} 1988		
		2 A g^{-1} 2540	1400 cycles 59.2%	
		5 A g^{-1} 2334	400 cycles 44.1%	
SiOx/CHSs	74-84	0.1 A g^{-1} 1078.7	100 cycles 60%	*Adv. Funct. Mater.*, ***2021***, *31, 2101145.*
		1 A g^{-1} 550	1000 cycles 85.3%	
SHCM/NCF	55	0.2 A g^{-1} 1960	300 cycles 96.9%	*Adv. Funct. Mater.*, ***2021***, *31, 2101487.*
		0.5 A g^{-1} 2200	530 cycles 96.9%	
		1A g^{-1} 1676.7	800 cycles 86%	
Si/CNF/G	42	0.1 A g^{-1} 1270.1	50 cycles 91%	*Nano Energy*, ***2014***, *6, 27.*
SiO_2/Sb@CNF		0.2 A g^{-1} 775	400 cycles 90.3%	*ACS Nano*, ***2018***, *12, 3406.*
		0.5 A g^{-1} 688	400 cycles 83.1%	
		1 A g^{-1} 614	400 cycles 76.2%	

References

[1] M. Wang, W. Song, J. Wang, and L. Fan, *Carbon*, **2015**, 82, 337.

[2] R. Zhu, Z. Wang, X. Hu, X. Liu, and H. Wang, *Adv. Funct. Mater.*, **2021**, 31， 2101487.
